# Disease priorities and rapid diagnostics testing preferences among community members in KwaZulu-Natal, South Africa: a formative qualitative study

**DOI:** 10.1136/bmjopen-2025-104997

**Published:** 2025-11-19

**Authors:** Thandanani Madonsela, Lenika Naiken, Chanda Prudence Mwamba, Anjali Sharma, Patricia Maritim, Ayanda Tshazi, Meagan Bemer, Amber Lauff, Jennifer F Morton, Alastair van Heerden, Hilton Humphries, Paul K Drain, the DASH 1 study team

**Affiliations:** 1 Centre for Community Based Research, Human Sciences Research Council, Pietermaritzburg, South Africa; 2 Center for Infectious Disease Research in Zambia, Lusaka, Zambia; 3 Department of Environmental Health, University of Zambia, Lusaka, Zambia; 4 University of Washington, Seattle, Washington, USA; 5 Department of Global Health, University of Washington, Seattle, Washington, USA; 6 SYNAPSE Research Collective, Wits Health Consortium, Pietermaritzburg, South Africa; 7 Department of Psychology, University of KwaZulu-Natal, Durban, South Africa; 8 Division of Social and Behavioural Sciences, School of Public Health, Faculty of Health Sciences, University of Cape Town (UCT), Cape Town, South Africa; 9 Department of Epidemilogy, University of Washington, Seattle, Washington, USA; 10 International Clinical Research Center, Deaprtment of Global Health, University of Washington, Seattle, Washington, USA; 11 School of Medicine, University of Washington, Seattle, Washington, USA

**Keywords:** Public health, HIV & AIDS, Tuberculosis, Molecular diagnostics, Africa South of the Sahara, Community Participation

## Abstract

**Abstract:**

**Objective:**

To explore and understand the disease priorities and preferences for rapid diagnostic testings (RDTs) among community members and stakeholders.

**Design:**

Qualitative study using focused group discussions and in-depth interviews. Thematic analysis was applied to identify themes of disease priorities and RDT preferences.

**Setting:**

uMsunduzi Municipality, KwaZulu-Natal, South Africa.

**Participants:**

49 community members and five community stakeholders were recruited through a combination of convenience and purposeful sampling using community events and meetings.

**Results:**

Participants prioritised both communicable diseases (HIV, tuberculosis) and non-communicable diseases (diabetes, cardiovascular disease, hypertension and cancer), aligning with national health priorities. They supported RDTs for early diagnosis and home-based testing to mitigate barriers to accessing diagnostic care. A need for post-test support, such as digital support tools, was also highlighted.

**Conclusion:**

Community perspectives highlighted a demand for accessible, rapid and decentralised diagnostic tools for high-burden diseases in KwaZulu-Natal. RDTs have the potential to improve health outcomes and reduce health disparities through improved access to diagnostic healthcare services. The community members are potential end users of RDTs, especially in resource-constrained settings. Therefore, their perspectives should be considered in the development and implementation of RDTs to enhance acceptability and public health impact.

**STRENGTHS AND LIMITATIONS OF THIS STUDY**

- This study captures the community perspective on disease priorities and preferences for rapid diagnostic tests (RDTs) at a time when more RDTs are being developed. Offering potential end-user insights.
- The use of both focus group discussions and in-depth interviews allowed for triangulation of data and, thereby, for rich qualitative findings.
- The community perspectives are based on a small sample size of community members residing in KwaZulu-Natal, limiting the generalisability.

## Introduction

 Similar to other countries in sub-Saharan Africa, South Africa faces a high burden of both communicable and non-communicable diseases (NCDs).^1^,^3^ In this region that is already battling infectious diseases such as HIV, tuberculosis (TB) and malaria, the increasing prevalence of NCDs has emerged as a major public health concern.^1^,^33^ In 2021, NCDs such as cardiovascular disease, diabetes and hypertension accounted for roughly 49% of all deaths, while communicable diseases, including COVID-19, HIV and TB, accounted for roughly 42% of all deaths in South Africa.^2^ This coexisting burden of disease, exacerbated by the rise in NCDs,^4^,^6^ poses a challenge to the healthcare systems that are already constrained.^1 7^ There is an urgent need for innovative and effective healthcare solutions to address these intertwined public health challenges.

Rapid diagnostic tests (RDTs) are one of the key strategies to address these public health challenges. RDTs have significantly improved access to testing services for conditions such as COVID-19, HIV, malaria, among others, allowing for decentralised disease testing and diagnosis.^8^,^13^ RDTs have been shown to improve health outcomes through timely diagnosis.^814^,^16^ They have been instrumental in epidemic surveillance and control^14 17^ and monitoring of existing NCDs,^5 17^ providing actionable results promptly. They also enhance engagement with healthcare services and early treatment initiation.^13 18^ Not only are they readily available in health facilities and communities,^8 10 15 17 19^ but they are also acceptable,^18 20^ accurate, safe and effective,^10 21^ and cost-effective, especially if integrated with routine care.^22 23^

In sub-Saharan Africa, the majority of available and in-use RDTs use the lateral flow immunoassay platform.^9 15 17^ This technology is the simplest, cost-effective and has been readily used in diagnostic tests, mainly for infectious diseases such as HIV, TB, COVID-19, sexually transmitted infections (STIs), influenza and malaria.^924^,^26^ In contrast, RDTs for NCDs are less common, with glucose monitoring and pregnancy tests being the most commonly available and used.^5 15 17 19^ RDTs for screening for kidney disease and high cholesterol have been described.^19 22^ Emerging technologies under development for infectious diseases, including molecular platforms such as loop‐mediated isothermal amplification^15^ and microfluidics-based platforms^9 15^ among others, their details are beyond the scope of this paper.

Despite these advances, RDT development has largely been shaped by diagnostic accuracy, integration into healthcare systems and cost considerations.^910 15 17 19 23^,^27^ Much less attention has been given to the perspectives and preferences of community members who are the intended end users. The acceptance and preferences of the community members are essential for the uptake of RDTs and continued use.^10 18 20^ Without insight into how communities prioritise diseases for testing and what is important to them about RDTs, there is a risk that RDTs may fail to achieve the intended public health benefits or even worsen health disparities.^19 23^

This qualitative study sought the perspectives of community members and stakeholders on priority disease and their preferences regarding RDT in KwaZulu-Natal (KZN), South Africa. By exploring the perspectives of the potential end user for RDTs, the study provides insights to guide the development and roll-out of RDTs in high-burden and resource-constrained settings.

## Method

### Design and period

This formative qualitative study was conducted as part of a larger, Digitally-Facilitated Access to Self-Care and Health Services in Low- and Middle-Income Countries (DASH) study. The DASH study aimed to understand the knowledge, attitudes and preferences of stakeholders and community members around self-testing for communicable and NCDs, as well as to evaluate the acceptability and feasibility of implementing a digitally-facilitated intervention for home-based self-testing. This was a multisite, multicountry study conducted in Kenya, Zambia and South Africa. In South Africa, the study took place from May 2023 to August 2023. The specific focus of this manuscript is community members’ and stakeholders’ perspectives from South Africa. The main findings of the larger DASH study are yet to be published.

### Setting

The study took place in the Greater Edendale and Vulindlela areas that are part of the uMsunduzi Municipality, a subdistrict within the uMgungundlovu District in KZN, South Africa. uMsunduzi includes urban, rural and periurban areas and has a population of over 600 000 people. The community has a high burden of HIV, STIs, early pregnancy and NCDs. Data was collected from rural and periurban communities within the Greater Edendale and Vulindlela areas.

### Study population and recruitment

Community members were recruited using convenience and snowball sampling, whereas community key stakeholders were recruited using purposeful sampling. We defined community members as individuals who reside in that community and spend more than four nights per week in the households of the study community. We defined community key stakeholders as individuals with a leadership or public service role, such as local political leaders, religious and traditional leaders and traditional healers.

The community key stakeholders were purposefully sought out by the study team in the communities where the study was being conducted. The stakeholders who agreed to participate were enrolled in the study. We conducted community drives in busy communal areas, including taxi ranks and shopping areas, to recruit community members. Individuals from the community were also referred to the study team by other participants.

The following eligibility criteria were applied to select participants: (1) a person over the age of 16 years, (2) residents of the study community, (3) who could read/write in one of the specified languages and (4) able and willing to provide written informed consent, or assent if aged 16–17 with a waiver for parental consent as applicable, were eligible for in-depth interviews (IDIs) and focus group discussions (FGDs).

### Public Involvement

Participants were not involved in designing and conducting the study. The community advisory board gave recommendations for the study implementation, including methods of recruitment and identifying the appropriate community for the study. The local authorities, as community gatekeepers, were engaged and expressed their support prior to the study implementation. Participants, through snowball sampling, referred other community members to the study team for study participation. Participants will be involved in data dissemination through community-based events and stakeholder engagements.

### Study procedures

Written informed consent was obtained from all participants who participated in data collection. Eligible participants were enrolled to participate in either the FGDs or IDIs to elicit feedback on disease priorities for testing and preferences of rapid test diagnostics, from community members and community stakeholders. FGD participants were mixed in terms of gender and age categories so that each FGD had representation across ages and genders. Community stakeholders only participated in the IDIs.

All study procedures were conducted by trained qualitative data collectors, aided by a pretested topic guide (AT and team). Qualitative IDI and FGD data were first captured on audiotape; thereafter, they were transcribed and translated into Microsoft Word documents. The demographic information for each participant was captured on the electronic data capture, REDCap V.13^28 29^ hosted by the University of Washington. Memos and notes from each IDI and FGD were captured on Microsoft Word by the qualitative data collector.

### Analysis

Data analysis was conducted by trained and experienced personnel on qualitative research. It involved reviewing memos and notes from the interview or discussion, transcription and translation of audio-taped data from isiZulu to English (AT and team). The transcribed and translated data underwent quality checks and assurance to ensure the meaning was preserved (AT and team). All transcriptions were deidentified before analysis.

Data was analysed using a thematic framework analysis. Using a preliminary codebook of the framework and concepts based on the study objectives, the transcripts were jointly coded by two qualitative researchers (CPM, PM) with NVivo V.12.2 (QSR International, NVivo (V.12.2) (Software). Melbourne, Australia: QSR International; 2018). The two researchers jointly coded the transcripts to keep the codes consistent. Discrepancies were discussed between the researchers until a consensus was reached. Through iterative reading of the textual data, additional codes and emerging themes were identified, and the codebook was amended as necessary. Excerpts regarding disease priorities and preferences for RDTs were extracted into Microsoft Word for synthesis (CPM, PM). The codebook is available as an online supplemental file 1.

The priority diseases and preferences for RDTs were identified from participants’ narratives from the FGD and IDs. We ensured the validity and reliability of our findings through triangulation, integrating the two data sources. For priority diseases, we coded and extracted excerpts for diseases that were either repeatedly mentioned across participants or where, in the FGD, a consensus was reached. This ensured that the listed diseases reflect a shared perspective rather than an isolated mention. References to diseases for which participants preferred to have RDTs for quick results and for self-testing were also coded, and relevant excerpts extracted. Using thematic analysis, these codes were grouped into broader themes. Participants did not review the analysis. For this manuscript, one researcher reviewed the excerpts and the organised codes against the transcripts to ensure context and accuracy (TM). The themes are outlined on table 1 of results.

**Table 1:** Outline of themes used during analysis

Theme and subthemes	Description
Disease priorities Common diseases Priority diseases/conditions for testing	Diseases or health conditions that are considered key or common in the community by community members/stakeholders or that the community members consider important for testing in general.
Preferred RDTs	RDTs that are felt to be suitable for inclusion in the intervention package to be used at home, in the community or for self-testing.
Priorities for rapid diagnosis	Description of top/key diseases/health conditions that should be prioritised for rapid testing in the communities.
Priorities for self-testing	Description of the top/key diseases that should be prioritised for rapid self-testing in the communities.

RDTs, rapid diagnostic tests.

## Results

A total of 54 community members participated, including 49 community members and five community stakeholders. 29 and 25 community members participated in the IDIs and FGDs, respectively. Table 2 shows a demographic summary of the study participants. The study participants were composed mainly of community members (90.7%) and females (53.7%). There was an overall high unemployment rate (66.7%) in this study sample.

**Table 2:** Participant demographics

	Type of qualitative method		
	IDI	FGD	Total
Characteristics	n=29	n=25	N=54
Age range (years)			
16–17	6 (20.7%)	1 (4.0%)	7 (13.0%)
18–24	5 (17.2%)	7 (28.0%)	12 (22.2%)
25–34	7 (24.1%)	4 (16.0%)	11 (20.4%)
35–44	3 (10.3%)	6 (24.0%)	9 (16.7%)
45–54	4 (13.8%)	3 (12.0%)	7 (13.0%)
55–64	3 (10.3%)	3 (12.0%)	6 (11.1%)
>65	1 (3.4%)	0 (0.0%)	1 (1.9%)
Chose not to share	0 (0.0%)	1 (4.0%)	1 (1.9%)
Male. n (%)	12 (41.3)	13 (52.0)	25 (46.3)
Female. n (%)	17 (58.6)	12 (48.0%)	29 (53.7)
Community member role, n (%)			
Traditional healer	3 (10.3)	0 (0.0)	3 (5.6)
Ward councillor	1 (3.4)	0 (0.0)	1 (1.9)
Community stakeholder (unknown)	1 (3.4)	0 (0.0)	1 (1.9)
Community member	24 (82.8)	25 (100)	49 (90.7)
Occupation, n (%)			
Employed	3 (10.3)	2 (38.0)	5 (9.3)
Unemployed	17 (58.6)	19 (76.0)	36 (66.7)
Self-employed/small market trade	4 (713.8)	1 (4.0)	5 (9.3)
Student	6 (20.7)	2 (8.0)	8 (14.8)
Chose not to share	0 (0.0)	1 (4.0)	1 (1.9)

FGD, focus group discussion; IDI, in-depth interview.

### Priority diseases or health conditions from the community perspective

The IDIs and FGDs enabled us to identify several diseases or health conditions that are a priority from the community perspective. These conditions were identified as a priority due to perceived prevalence and/or as important for testing in the community.

The conditions identified by the community can be broadly categorised into communicable diseases and NCDs. The communicable diseases elicited include HIV, TB, STIs, diarrhoeal diseases such as cholera, skin conditions such as smallpox, measles and mumps, common cold and influenza, COVID-19 and meningitis.

I think the important ones; the first would be for HIV testing because it looks like it’s the one with high rates of spreading in our community. (Community Stakeholder, IDI)

The NCDs elicited included cardiovascular conditions, hypertension, diabetes, musculoskeletal conditions such as arthritis and gout, epilepsy, respiratory diseases such as asthma and allergic rhinitis, cancer and mental health conditions. See online supplemental file 2 for excerpts.

### Rapid diagnostic test preferences for rapid diagnosis

The following conditions consistently emerged as important conditions for rapid diagnosis using RDTs: HIV, TB, hypertension, diabetes, COVID-19 and cancer. As demonstrated by responses from FGD 1, when asked what conditions are important for rapid test results, participants listed various conditions.

P1: HIV

P2: It’s TB

P3: Diabetes

P4: BP (Blood pressure)

P2: It’s cancer

(Community Members, FGD 01)

Participants were subsequently asked to select the top three conditions they considered important for testing using RDTs. There was consistent consensus across participants that HIV and TB were part of the top three key conditions for RDT use. The third condition varied across participants, including diabetes, hypertension and COVID-19. In one FGD, while hypertension was considered to be part of the top 3, there was a group consensus that COVID-19 was more important than hypertension. This was attributed to the perceived acute and high mortality of COVID-19 in comparison to that of hypertension.

P2: Yes, Corona [virus disease 2019] … The worst part is that COVID is worse than BP [high blood pressure]. With BP, you can get medication and get treated. With Corona [virus disease 2019, you get injected [with vaccine], but still die. There are a lot of people who got injected with that injection but died. So, with BP you get medication but you don’t die, it helps you and heals you. (FGD-02, Community Members)

Other conditions that were mentioned, despite their low prevalence in South Africa, were malaria and mpox. However, participants expressed no specific preference or prioritisation for the use of RDTs for these conditions.

### Rapid diagnostic test preferences for self-testing

When participants were asked about preferences for RDTs for the purpose of self-testing in their communities, the following conditions emerged as the most priority conditions for self-testing: cancer, HIV, TB, diabetes, hypertension, pregnancy and STIs. Many participants reported that RDTs for self-testing would be both comfortable and easy to use at home. The stigma associated with HIV and pregnancy was frequently mentioned as a motivating factor for preferring home-based or self-testing: ‘I think what the people are most shy about is HIV/AIDS testing’ (Community Stakeholder, IDI).

Self-testing for HIV was consistently discussed. Reasons that emerged for HIV self-testing include timely diagnosis, timely treatment initiation and prevention of opportunistic infections, ‘…knowing your HIV status is very important so that even if you are positive, you can take your medication to minimise being infected by various diseases and end up being a person who is easily infected by [opportunistic] diseases anyhow’. (Community members, FGD 03). Also, women protecting themselves and their unborn baby was also mentioned as one of the reasons for HIV self-testing preference. ‘Firstly, women are breastfeeding, let us say that, so if it happens that you have given birth to a baby, and then you become intimate with someone, you didn’t have it, whereas they have HIV. In that time you are still breastfeeding, who will that affect at the end, it will affect the child. So, it is better to have it [HIV self-test] with you, so if you know that you are sexually active but you do not use protection or you do not take these things [PrEP] that are taken to protect [against] HIV, you can keep testing yourself in order to always protect your baby’. (Community members, FGD 04).

While many participants preferred the use of RDTs for conditions such as HIV and others, to mitigate stigma associated with visiting the clinic, they also highlighted the potential need for counselling support. As one community member explained, ‘… testing at home please, can I put it like this, it’s good, [and] it’s bad because isn’t that you will be counselled after you have found out that maybe you are HIV positive? You will need someone who will help you understand this clearly. Let’s say I found out again that I am HIV positive, this thing is now a burden for me, how will I counsel myself? I need someone who will help me basically, so it’s good to test at home on the other hand its bad’. (Community members, FGD 01*)*

Self-testing for conditions such as pregnancy, diabetes or hypertension was also preferred not only for early detection but also potentially to prevent condition-related complications. ‘… This [is] because adolescents get pregnant so they have to get help fast; and then this one for grandmothers such as diabetes and other diseases that affect the older people which could help them since an old person need to be tested as in now! Because one might just fall and you find that the other one gets sick whereas when you notice fast that diabetes [blood glucose] is elevated, you would be able to [help] her fast by giving first aid at home’. (Community Stakeholder, IDI)

In addition, participants mentioned that the availability of testing support, such as a mobile or digital tool offering guidance and support during the self-testing process, would further enhance their preference for using RDTs in the home setting. ‘[An app] will help a lot because once you have tested and find out that you are positive, you will get instructions telling you to go to the nearest clinic…’ (Community Stakeholder, IDI)

## Discussions

### Disease priorities and community perceptions

Priority diseases and conditions identified for testing reflect the high burden of both infectious and NCDs in the communities.^4 5^ Notably, in line with national health priorities, participants identified HIV, TB and diabetes as key conditions of concern.^30 31^ This is also aligned with the reported prevalence of these conditions in the province of KZN, South Africa.^4 5^ This alignment between the community-identified priorities and national health priorities underlines the importance of community input to form relevant health interventions.^15 30 31^ The growing concern from health authorities over conditions such as cancer and mental health disorders^1 30 32^ is reflected in their repeated mention by participants as priority diseases, even though they may not have the same immediate prioritisation as HIV or TB.

### Rapid diagnostic test preferences

The community preference of RDTs for diseases, such as HIV, TB and diabetes, highlights that early detection and management of such high-burden diseases^1 4 6^ is also important to the community members. Second, the preference for home-based testing to mitigate stigma, ensure privacy and autonomy highlights the potential of RDTs in enhancing access and uptake of diagnostic services and improving health outcomes.^5 8 14 15^

Indeed, the community preferences align with the continued global and national effort to decentralise healthcare services as far as possible through community-based and home-based testing.^1316 22 33^,^35^ For instance, the WHO endorsed HIV self-testing as a means of reaching the undiagnosed.^34^

While some of the participants mentioned RDTs for malaria and monkeypox, these conditions are not highly prevalent in South Africa. It remains important that while RDT use for various conditions may be acceptable to communities, the selection of RDTs should still be guided by local epidemiological data to ensure their relevance and cost-effectiveness.

### Stigma and support for self-testing

Participants highlighted that home-based testing would also need follow-up support, especially for individuals who test positive. This highlights the need for seamless integration of RDT use for self-testing with existing healthcare services such as counselling and support provision, either through healthcare providers or digital tools like telehealth or mobile applications.^4 5 7 22^ Companion mobile health technology would be able to support, advise and connect people with health services, especially for conditions such as HIV, in which post-test counselling is needed. Thus, technology can play a potential role in improving the self-testing experience.^36^

### Implications for health policy and interventions

The community’s preference for use of RDTs indicates an opportunity to scale up RDTs implementation for self-testing, particularly for conditions where RDTs are readily available, such as HIV, pregnancy and diabetes.^15 17 19 34 35^ The disease priorities and RDT preferences identified by the community underscore the need for accessible, rapid and decentralised diagnostic tools that are integrated with the existing health systems. As far as possible, the integration of screening and diagnosis for communicable diseases and NCDs is recommended. NCD and communicable disease screening and diagnosis at the community level is feasible, acceptable, impactful and efficient.^7 10 13 14 16 19 22 23 36^

### Limitations

Notably, the unemployment rate among participants in this study was 66.7% which is much higher than the national (32.1%) and provincial (29.5%) unemployment rates in the same year.^37^ This and the study sample size limit the generalisability of our study findings to the broader population. A larger sample size with systematic sampling is recommended for future studies.

## Conclusion

In conclusion, community perspectives highlighted a demand for accessible, rapid and decentralised diagnostic tools for high-burden diseases in KZN. RDTs have the potential to improve health outcomes and reduce health disparities through improved access to diagnostic healthcare services. The community members are potential end users of RDTs, especially in resource-constrained settings. Therefore, their perspectives should be considered in the development and implementation of RDTs to enhance acceptability and impact.

## Supplementary material

10.1136/bmjopen-2025-104997 online supplemental file 1

10.1136/bmjopen-2025-104997 online supplemental file 2

## Data Availability

All data relevant to the study are included in the article or uploaded as supplementary information.
