# Supplementary material for: Disease priorities and rapid diagnostics testing preferences among community members in KwaZulu-Natal, South Africa: a formative qualitative study
Source: BMJ Open. 2025 Nov 19;15(11):e104997. doi: 10.1136/bmjopen-2025-104997 (PMC12636905; doi:10.1136/bmjopen-2025-104997)
Supplement: online supplemental file 1 [file bmjopen-15-11-s001.pdf]

Digitally-Facilitated Access to Self-Care and Health Services in Low- and Middle-Income Countries (DASH): Qualitative Study Codes

Codes

Name	Description	Files	References
Acceptability of Self testing using RDTs	A multifaceted construct that reflects the extent to which people delivering or receiving an intervention consider it to be appropriate based on anticipated or experienced cognitive and emotional responses to the intervention.	0	0
Affective attitude	How an individual feels about the intervention.	16	53
Benefits	Perceived benefits acquired through taking part in the interventions	17	44
Burden	The perceived amount of effort that is required to participate in the intervention	12	21
Ethicality	The extent to which the intervention has good fit with an individual's value system	17	48
Excluded RDTs	RDTs that are not felt to be suitable for inclusion in the intervention package to be used at home.	1	1
Follow up steps after testing		14	26
Individuals to collect tests and medications from		1	1
Intervention coherence	The extent to which the participant understands the intervention and how it works I.e whether they use RDTs and how often they use them at home	14	27
Negative results	Coping strategies to deal with negative results	4	4
Opportunity cost	The extent to which benefits, profits, or values must be given up to engage in the intervention	2	4
Perceived effectiveness	The extent to which the intervention is perceived to be likely to achieve its purpose	14	32
Positive results		4	5
Preference on receiving instructions	What would be your preferred way to receive information on your test results – in person, via	3	4

Name	Description	Files	References
	phone call? Via video call? Via WhatsApp, over SMS? From an app?		
Preferred RDTs	RDTs that are felt to be suitable for inclusion in the intervention package to be used at home	16	26
Self efficacy	The participant’s confidence that they can perform the behaviour(s) required to participate in the intervention	15	32
Testing locations	Preferred venues for self testing	18	41
Testing times	Preferred time for self testing depending on the disease or RDT	0	0
Priority diseases		0	0
Available digital interventions	Description of digital health interventions that are currently available in the country.	4	7
Available RDTs including self testing kits	Description of rapid diagnostic tests that are currently available in the country.	9	20
Community resources needed for self testing		15	30
Experience of introducing using digital interventions	What has been the experience of introducing the digital interventions in the country	4	5
Experience of introducing using RDTs	What has been the experience of introducing & using the RDTs in [country]?	4	7
Other conditions for home testing		0	0
Overall disease priorities	Which are the diseases that the country will focus on for the next 5 years?	0	0
Community's role in promoting health		4	5
Disease priorities for the next five years		6	6
Recommended improvements		0	0
Successful strategies used		3	4
Priorities for rapid diagnosis	Description of top diseases that should be prioritized for rapid testing in the communities that you serve, and why.	15	26
Priorities for self testing	Description of top diseases that should be prioritized for rapid testing in the communities and why.	2	2
