# Supplementary material for: Disease priorities and rapid diagnostics testing preferences among community members in KwaZulu-Natal, South Africa: a formative qualitative study
Source: BMJ Open. 2025 Nov 19;15(11):e104997. doi: 10.1136/bmjopen-2025-104997 (PMC12636905; doi:10.1136/bmjopen-2025-104997)
Supplement: online supplemental file 2 [file bmjopen-15-11-s002.pdf]

Disease priorities and rapid diagnostics testing preferences among community members in Kwa-Zulu Natal, South Africa: A formative qualitative study

Appendix 2: Excerpts from participant transcripts

Files\\DASH IDI, Community Stakeholder, Sweetwater (South Africa Rural Site) 13 July 2023 (2) - § 3 references coded [4.61% Coverage]

Reference 1 - 1.31% Coverage

I: Okay. Uh, what test kits must be prioritized for your people in your community?
P: Maybe, can you please explain in detail?
I: Maybe eh, isn't that the testing kits are different?
P: Mm.
I: Just like… let's say you go to pharmacy and buy a kit for pregnancy test. That's still a kit. Then you buy a kit to test for TB, maybe buy a kit to test for HIV. Those types of kits for different diseases.
P: I think the important ones; the first would be for HIV testing because it looks like it's the one with high rates of spreading in our community.
I: Yes.
P: Our community is dying now and again due to HIV/AIDS. And then, the second one would be for TB.

Reference 2 - 2.29% Coverage

I: TB.
P: Because HIV is likely to go hand in hand with TB.
I: Okay.
P: That of pregnancy test, yes but I fear it for one reason because it's like we are giving our children freedom to do whatever they want.
I: Mm.
P: Because she will wake up in the morning after sleeping with a man and found out that, they are positive. They turn to go there in the streets, there are [cross talk] those who conduct abortions in the streets. And by the time the girl gets married useyigxaba lesalukazi (damaged goods/loose) because they have had abortions more than 20 times.
I: Mm.
P: Yes, but we can't stop them from receiving these because it's their right. But I don't recommend that.
I: You don't recommend it.
P: Because it's killing our nation.
I: Mm. Sometimes I hear that having an abortion can bring bad luck.
P: Mm.
I: Maybe, things of that child are not going well.
P: Every time. Tradition says "the child that you have aborted, the time you thought you aborted it and got rid of it, their spirit remains alive"
I: It's alive.
P: Until you reach the age where you must perform a traditional ceremony for that child.
I: It's old!

Reference 3 - 1.01% Coverage

: Okay sir, eh… I heard you mentioning HIV, and what else, TB and what else?
P: TB, Diabetes, BP.
I: Eh, maybe in order of importance of these diseases. Which three rapid diagnostic tests should be available for the community? Say like, this one is needed in the community and then maybe number two and so on.
P: I think the ones who are leading right now, three of them. Maybe, it would be HIV/AIDS that's number one.

I: Yes.
P: Number two, it would be diabetes and then number three would be BP.

Files\\DASH IDI, Community Stakeholder, Traditional healer, Sweetwater (South Africa site) 04 July 2023 PID 496602907 (2)-QC - § 5 references coded [14.81% Coverage]

Reference 1 - 3.68% Coverage

I: Okay. Umm [pause] which rapid test kits do you think should be prioritized for the people in your community, and why? So, maybe the kits, there is one for HIV, Diabetes kit, BP, umm, Malaria, pregnancy, and STIs. So, which rapid testing kits do you think should be prioritized for the people in your community?
P: [clear throat] It is for HIV, Diabetes, there is also the one you didn't mention, but maybe you did mention it I might have missed it, the one for cancer.
I: Oh, cancer!?
P: Yes, uterine cancer for women. I don't know about it because it won't be able to be conducted at the hall or maybe if there would be those who would come to the clinic and have a reserved space, I think there are many people who would like to go there and test because most of the time when people are visiting the clinic, normally it is announced at the clinics that let's do pap smear, others might consider the issue of time because they have been there since the morning and have stories, I won't make it. Then they take their medication, leave, and say they will see some other times. Maybe even on the next time, whereas if it could be brought near, like in the hall, and have a mechanism as to how it can be done. But also, what might a disadvantage to others, things happen during weekdays, and you find that people are complaining that they won't make it because they are working, preferably if it was during weekend. All clinics do not operate on weekends, except XXX where they operate even during the weekend, it does operate on Saturdays and Sundays, although not all the sections operate like a pharmacy, at the pharmacy you get the medication from the doctor inside and the pharmacy by then, is closed.

Reference 2 - 2.74% Coverage

I: Okay, and HIV and diabetes? Why do you think, umm, they should be prioritized?
P: HIV and Diabetes, I can say that HIV, what would make it a priority is that people are dying, you find that someone tested and have that feeling that they have nothing, they think about that while they feel well whereas this thing is killing them inside and they also do not prevent in believing that they are positive, they do not abstain from having sex to protect themselves, he keeps on doing what he does. He further approaches various women and sleeps around, spreading this disease. While he is spreading this disease, there are a lot of people who have been infected because of him. The problem is that men are so difficult, that they do not want to visit clinics to test themselves, and with diabetes, it happens that someone has diabetes and they do not follow proper procedures of how to take their diet, another person might be told that if you have diabetes, you should not eat salty diets, and you find that person uses salt, cooking oil, the sugar levels are high, to a point where they have to get an injection. I normally hear that once you are bound to have an injection, it is very dangerous.
I: Yes, because your body can't [cross talk].
P: It is uncontrollable.

Reference 3 - 5.42% Coverage

: Yes, you now have to be assisted by an injection.
P: Yes, you now have to be assisted by an injection all the time. Then, there is something else that we missed, TB. TB is also dangerous in terms of, for example, let's say at home we are many under a single roof. This person has TB, and if this person has TB, maybe this person has a cough, by the time this person coughs, there is no way they can protect themselves, the disease spreads and we will all catch it in this household. Also, if this person is inside the house, some people might think they are discriminated against, whereas that is not the case, they should be protected in their space and have their own utensils that will be distinguished from the rest of everyone until they recover because

children are easily infected with it. Not unless as a family, you go and notify the clinic that there is a family member who is like this and be able to get medication to protect you. Also, that pill can be used, but if the person is still with you, chances might be there won't be any difference. For example, if TB intensifies, there is someone who visits the clinic and they give them the necessary medication, you will take this pill for 6 months and come back again to check how is the progress. You find that someone doesn't take their medication, they lie to you as their guardian, they are sleeping and you keep on giving them their medication and they decide not to take their pills because you are distracted by something, you will think they have drank it whereas they have threw it under the bed and drank only water and you will think everything is going fine. After some time, and you realize that there is no difference, how come because they take their medication? Until someone gets extremely sick to a point where they can't even walk, and when they get to the hospital, they tell them, what do you call it, MDR? That means this person is extremely sick and can never be assisted. Maybe someone might be lucky, get assisted, and be saved by the doctors until they become better and they get back home and they are told to use their medication for a certain period of time, and others go against that because they feel well and they put their medication elsewhere. Another thing that is painful about TB is that it is the deadliest disease but its danger can be controllable because you do not live with it for the rest of your life, it's just a certain period of time that is specified for you to use it and then after that, you will be okay.

Reference 4 - 1.29% Coverage

I: Okay. Umm… In order of priority and importance, what are the top three rapid tests that should be made available to communities from among those that you mentioned?
P: Three?
I: Yes, three, maybe the ones you think are the most important diseases that are supposed to be tested in your community?
P: Ey, where I think it should be tested would be at the hall, where the tests could be conducted, that could be a safe space and it could be rapid, a lot of people can be assisted.
I: Okay. Maybe the conditions that should be tested?
P: HIV, Diabetes, TB, mmm, HIV, Diabetes, TB, and BP.
I: BP?
P: Yes.

Reference 5 - 1.68% Coverage

I: Okay. Uh, which tests do you think the people in your community would be comfortable to self-test in their homes or venue of choice within the community, and why?
P: It is HIV, and what would make them happy is that they do not like to attend clinics because they are scared. They are scared because they will be known what disease they have because even others do not believe that HIV is now a disease that is no longer taboo, and you won't be concerned if you find that you are positive. You won't be scared because it is now a disease that is normal. The only thing would be, you should avoid a lot of things and do what is required. You are able to live a long life, and you live a healthy life where you are not seen you have a disease because you follow the necessary procedures.

Files\\DASH IDI, Community Stakeholder, Ward Councilor, Sweetwater (South Africa Rural Site)14 July 2023 PID 49-66-059-0-6 -QC - § 2 references coded [4.48% Coverage]

Reference 1 - 2.52% Coverage

I: Okay maybe which err rapid test kits do you think should be prioritized for the people in your community, and why?
P: Maybe it is the one of HIV/AIDS because it is the disease that always worries us and then pregnancy. This because adolescents get pregnant so they have to get help fast and then this one for grandmothers such as diabetes and other diseases that affect the older people which could help them since an old person need to be tested as in now because one might just fall and you find that the

other one get sick whereas when you notice fast that diabetes has elevated, you would be able to her fast by giving first aid at home.

Reference 2 - 1.95% Coverage

I: Mm okay err in order of priority, what are the top 3 rapid tests should be made available to communities from among those that you mentioned?
P: Err the one that I put first is the one that is used by grandmothers because a person can just fall and anything can happen since it is life threatening while the others such as the HIV can be the second one and pregnancy becomes the third option. So, the first one is life threatening because it is disease where anything can happen at err that time.

Files\\DASH_IDI Community Health Care Worker, Sweet Water (South Africa Rural site) 28 JUNE 2023 PID 49-66-044-0-6 - Copy-QC - § 1 reference coded [3.36% Coverage]

Reference 1 - 3.36% Coverage

I: Okay, so which ones do you think should be prioritized for home-based, you have mentioned them, can you please repeat?
P: I said it's HIV, Diabetes, BP, and cancer. [pause]
I: Okay.
P: Yes.
I: All right, so, in order of priority, which top three from the ones you mentioned, err, that you think should be prioritized, should be a priority for rapid testing in the community that you serve, and why from the ones you mentioned?
P: [sigh] TB!
I: It is TB.
P: Yes. Then, it is HIV.
I: HIV.
P: [pause] And cancer.
I: And cancer.
P: Yes.
I: Why? Maybe we could start with TB.
P: Mhm.
I: Mhm.
P: I start with TB because… in fact, TB is deadly.
I: Mhm.
P: Yes… Whereas if someone was quickly diagnosed with TB, they can be treated.
I: Okay.
P: And get assisted.
I: How about HIV?
P: HIV is very problematic because the person who infected another one just spreads the disease everywhere. This one is just doing nothing, for example, as women, you don't do anything waiting for your husband whereas the disease is eating you inside.
I: Mhm.
P: Yes.
I: Okay. You also mentioned cancer. What about cancer?
P: And cancer because it is also, I think cancer is very dangerous because it normally doesn't appear, and you find that the disease is found whereas it has been spread all over the body and you find that they won't be further assisted and they die. Whereas we normally teach them if there is something you feel in your body, it might be a pimple or you usually check your breasts and check whether is there something you feel, hurry and visit the clinic. You find that people don't care about that. Maybe they will say it is something that eventually, will be all right whereas it is something that will end up killing them.

Files\\DASH_IDI Community Health Care Worker, Sweet Waters (South Africa Rural site) 29 JUNE

Disease priorities and rapid diagnostics testing preferences among community members in Kwa-Zulu Natal, South Africa: A formative qualitative study

2023 PID 49-66-028-0-3 QC - § 2 references coded [1.88% Coverage]

Reference 1 - 0.64% Coverage

I: Mmh, okay erh you have mentioned the priority diseases and said its TB, diabetes, HIV.
P: Yes.
I: Erh so, erh and you also mentioned…
P: And pregnancy.
I: Pregnancy, yes.
P: Yes, pregnancy.
I: Oh, and epilepsy.
P: Yes.
I: Mmh, epilepsy [writes on pad].
P: Especially, do you see maybe at the clinic, there aren't that many males, there are a lot of women.

Reference 2 - 1.25% Coverage

I: Males don't go there, erh so what 3 diseases , you see, just 3 that you prioritize, that you think that no these must be prioritized with this rapid home testing, you see the one that you can yourself. Which ones would you prioritize?
P: TB,
I: TB…TB.
P: HIV.
I: Oh, HIV, and the last one?
P: It's diabetes
I: Diabetes [writes on pad].
P: Because a granny may have a sore [inaudible segment]
I: Yes, they would tell themselves no…
P: It won't get better, whereas it's spreading, you'll find that at the hospital, their leg will be amputated.
I: Whereas if maybe there was something to test yourself at home with, and be able to test themselves and see that no I have diabetes and then rush to the clinic.

<Files\\SOUTH AFRICA\\FDGs Community members\\Dash FGD 01 Community Members Sweet Waters (South African Rural site) 15 May 2023(PID 496600202,496600403,00600,496600104,496600508) Transcript-QC (1)> - § 8 references coded [23.31% Coverage]

Reference 1 - 8.68% Coverage

F: Oh, alright, we have counted 17 illnesses, uuh with these 17 illnesses please may I ask which ones do you think we need to be able to test at home and to get quick results? If we test at home

P2: P2 [silence]

F: Okay, just a minute [silence] okay we will start with the diseases that we see the need for testing with RDT (Rapid Diagnostic Test) so that we get quick results [mumbling] that we see the need for quick testing, to get quick results

P1: HIV

F: HIV, mmh okay

P2: It's TB

F: TB

P3: Diabetes

F: Diabetes

P4: BP (Blood pressure)

F: Okay, uhm HIV, TB, Diabetes what was the other one?

P4: BP

F: BP, oh I had added it

P2: It's cancer

F: Cancer,

P4: Epilepsy

F: Okay, [silence] mmh can we carry on? Is it alright? What are we saying about STI's?

P4: Yeah

P5: An STI needs a clinic

F: Okay, now we were asking, do you remember the RDT I said it can produce quick results, now I will be asking about testing at home, it's a self-test, so we don't see an STI fitting in here? In quick results? Rapid diagnostic tests do you remember me talking about them? Okay, here we are going to, what we think will be nice if we could test ourselves with these diseases, those that we have named. We talked about common diseases, we spoke about diseases that need quick testing now we are talking about uhm diseases that will require you to test yourself. From their importance we will list them so that they become three.

P4: Over there, where there are ones that are quick, wont a pregnancy test fit in?

F: With these?

P4: Yes, to the quick ones

F: Quick ones, okay so a pregnancy must come this side (writes on board) it wasn't on this side. Which ones do we think we would like to test ourselves?

P2: It can happen that we remove them from the RDT and bring them back here to where we had

F: Three is what I want

P2: Yes

F: From all of these, just three

P2: Just three?

F: That we see as the ones that have to be a priority for us to test at home

P2: Oh, at home, HIV for me is the first one

F: Okay

P5: I would say TB

F: TB

P1: Please may I ask, please can I ask, as it is said that you will test at home, will they give you a kit for you to do it yourself or will there be someone who will [mumbles]

F: As?

P1: As it is said that you will test at home, will they give you a kit for you to do it and then they go or is someone going to help you?

F: Yeah, that's why it is asked, when we say self-test that's why its asked but we will get there if we carry on with the questions where we're asking on how the kit is you see? For now, I would like to know maybe what you would like to test yourself with at home

P2: Oh, I'm not sure if cancer is important, some people don't know if they have it but It's important to test yourself at home, just to check yourself of cancer

F: Mmh cancer, okay so we see TB, HIV, Cancer?

P1: I think, okay please so that we can get clarity

F: Mmh

P1: You said if you test at home, they will bring the kit to you, be left alone and do it yourself?

F: Ideally, when they self-test it has to be something like that, so that you test yourself at home but if we continue with the questions, there is where I will ask you if you would like to test yourself or if you want have someone help you and so on, it's just that now I was just wanting to know what diseases you think that ey it is needed that I test for it at home so that I don't go to the clinic

P4: Diabetes

F: Diabetes

P4: Yes

F: Okay, so we have mentioned HIV, we also mentioned TB, we mentioned cancer, right? We said here, how many mentioned HIV? two? We also mentioned TB, how many of us mentioned TB? Did we mention TB?

P2: Yes

F: We even said [inaudible segment]

P2: Yes, we did mention it

F: Is it P5? Okay, we also said cancer, how many of us?

P2: Yeah, I said cancer

F: Two, maybe

P5: Diabetes can also be added

F: Diabetes?

P5: Yes

F: Is there anyone who agrees with diabetes?

P2: I heard you say …

F: We want, from the mentioned diseases I want to see three that we all agree on that these are the ones that we think we would want to test at home, yoh it's hot, I will faint [laughs] please lower it (heater) a bit I will faint, yes, I want us to all agree, isn't in your community, so, we can't say TB whereas one person mentioned it, with these diseases we are all mentioning these diseases, with these diseases we saw which ones need to be made fast, so that we can get [whispers] so that we can get quick results. When we have received quick results, which ones do we think that ey you see I want quick results and I want to test myself at home so we have to agree which disease that we know to be a problem near our homes and which ones do we want to receive being able to test at home before you go to the clinic, because it's the most common and sometimes I need to be able to test for it from the ones we have written [silence] okay maybe let me do it like this, how many of us said HIV?

P2: Erh, yeah, we will add HIV

F: So, we all agree with HIV?

All: Yes

F: We need to be clear, okay, and then with TB? I am looking from these

P2: With TB I can say that it's also dangerous because sometimes you would feel that your chest has a problem but you don't know what you have but what is important is maybe check yourself and see what you have, do you have that disease, I don't know if they will agree if we will add TB

F: No, I am asking from you, you will have to tell me if you think TB has to be there

P2: Yes, it has to

F: Okay, I am hearing three who agree

P2: Yeah, we all agree

F: For diabetes?

P3: Because cancer is a problem

P2: Yeah, it's dangerous

F: Oh, lets first maybe go with, do we all agree with cancer?

P3: Yes

F: Do we all agree with cancer?

P2: Yes, we agree

P1: We agree

F: [laughs]

P3: We agree, why am I saying this? I am saying this because of my brother who was a Doctor. He found out later that he had cancer, he was just living, we buried him in 2021

F: Mmh

P3: Yes

F: So, it’s something that you would like to test for at home?

P3: Imagine he was a Doctor, he didn’t know but he heals people who have cancer only to find that he has it, he didn’t feel it. They said it was too big, just like I was saying that it’s a problem with that

F: Oh, okay everyone, cancer?

All: Yes

F: Okay, BP? [murmuring] okay maybe let me do it like this, I must go like this, which three diseases do you think must be quickly tested because it’s like we want to see if this one agrees, this one disagrees, maybe P5 which three things do you think that these must be really tested at home?

P5: Okay, HIV, TB and then erh what is it called? Diabetes

F: HIV, TB, Diabetes, okay P4?

P4: Diabetes and HIV

F: Mmh

P4: And BP

F: And BP?

P4: Yes

F: Okay, we will start again, alright

P3: It’s Cancer and Diabetes

F: Cancer and Diabetes, okay the third one?

P3: Ey, and then it will be Diabetes

F: Diabetes, okay, alright P2?

P2: Oh, it is HIV, erh then Diabetes

F: P3 you didn't mention HIV, right? You didn't?

P3:No, I didn't, I didn't mention it

F: Okay, HIV?

P2: Erh eh, yeah HIV, TB and Cancer, it's the ones that are on this side

F: It's TB and Cancer, okay P1?

P1: All of the ones this side, HIV, TB and Cancer

F: Okay, HIV, TB, Cancer, okay here from what I see Cancer has five, HIV has four, Diabetes has three, that means TB is not here

P2: Yes, we will remove it

F: Okay, so it means its HIV, Cancer and Diabetes, okay thank you, alright then uhm we will now move forward, uhm [silence] so urh [silence] okay, so which other diseases need to be tested for? Others that we maybe didn't write here that will need for us to test for, maybe that you think of now that we didn't write here, other diseases, what are they that you think you would like to test ourselves? Even if you wrote it here, or if its not here

P4: Eczema

F: Eczema, okay, alright P4 said eczema, what else do we think we would like to test ourselves for? What are the others? That we ended up not writing in the self-tests? [paper sounds] there isn't anything else? That we think we need to test at home?

P1: No, there isn't

F: There isn't? okay, it's just eczema? Uhm, what about pregnancy? Don't we need a self-test so that we test at home?

P2: We can also add it

F: Pregnancy, STI's?

P5: Yes

F: P5 is saying that STI's we need to pour, and the rest of us? To test for ourselves, what are the rest of us saying? Sexually transmitted diseases?

P2: Yeah

F: If it's no, it's a no, if it's a yes then it's a yes

P2: I said I agree, STI's yeah

F: What are we saying, P4, P3?

P3: It can be added [mumbling]

P4: And then BP can be added

F: Must we also add BP?

P4: Yes

Reference 2 - 2.37% Coverage

Okay, we have pregnancy

P2: Yes, yes, yes

F: Okay, we have Covid-19, okay please can we speak loudly because I want it to be heard that I am talking and people are answering, it must not be as if I am removing you see?

P3: It's just that Covid-19 is a disease that quickly passed

F: Ooh, so what are we saying about it?

P3: We will no longer explain it

F: Okay, sir said we will no longer need Covid-19, what do we say?

P2: Oh no, we will take out Covid because ei

P3: It really didn't treat us well

F: Okay, the vote is against you, alright we have…we said will leave pregnancy, we have HIV?

P2: Yeah, we will put it

All: Yes

F: We will leave it, okay, okay let me say all of them so that I won't get to other ones …we also have diabetes, STI's, BP, Mental Health Screening, Physical health screening eerh yeah, which one do we want to add here?

P4: BP

F: BP

P4: Do we all agree?

F: Yeah, what do we say about it?

P2: Oh, oh, BP? Checking of the blood?

F: It's BP

P2: Oh [laughs] BP, yeah yeah, I agree with it because yeah sometimes you have to check how much your blood pressure is

F: P2 said he agrees with BP, and us?

P1: No, no I don't agree

F: No? okay, sir BP?

P3: I definitely agree

F: You agree? 2 for BP, is there anything else maybe? Let's first explore everything, STI's, diabetes

P2: Diabetes, yes

F: What do the rest of us say about Diabetes?

P1: We agree

P3: We agree

F: We agree, sister?

P4:Yes

F: Okay, BP, Diabetes and the rest? Here they are, they are in front of you. I am relying on you to tell me the ones you want

P2: [inaudible segment]

F: You have to talk loudly

P2: Oh, Physical Health screening yeah, I can say that its important to screen your body

F: Mmh

P2: Yeah, so that the scale will balance, all of that …just how you live, yeah

F: Guys, we are allowed to say no if you feel that the one he says you don't see it, its your right, you are also allowed to agree

P1: Oh, no

F: P1 disagrees

P5: P5 as well

F: Alright then so its testing…alright mental health, STI's, or the ones we chose are we alright with them?

P2: Mental Health screening, oh can it happen that maybe you say you have a child, maybe they don't know what is happening with their child, when it's still small you obviously can't see that the child is not alright, so it can happen that you check the child, just checking it at home

Reference 3 - 3.25% Coverage

P1: I think that needs people who have studied for it, yes you can see it but you can't check it yourself at home

F: Mmh, what are we saying with mental health? Mental health, sir?

P3: Ey I don't see it

F: Okay, I think we all agree that we will take BP, HIV, Pregnancy, Diabetes?

P4: Yes

F: Alright, then others we will put aside, okay then we have chosen from these that we

have chosen I will ask about them, we can all see right?

P5: Yeah

F: From them, we have two options, we have options for…thank you sir, would you like [silence] oh we have explained already why we chose diabetes, we can count it again, I don't think we mentioned it, why did we choose diabetes? Maybe with all of them, you can explain diabetes, pregnancy, HIV and BP

P1: Thats why we chose them to test at home?

F: Yes, mmh

P1: Okay urh P1, I think that there are people who wouldn't like how they live to be known, who wouldn't like if their results be known even by doctors

F: What are you talking about here, which disease?

P1: HIV

F: HIV, ooh, okay

P2: Oh, for diabetes

F: Mmh

F3: Sorry, I'm just suggesting, please may I ask if they can maybe say HIV and list everything, then move on to another disease when they are fine with HIV then they can move on to another disease and so on

F: Yes, lets continue with HIV, thank you [name mentioned] we can continue with HIV, That's why we chose it, why is it important for us to test at home [silence] okay P1 said that he thinks it's important to test for HIV at home because some people won't like it if their results are known. What about the rest of us, we all have chosen HIV that we would like to test for it at home, maybe we can bring reasons as to why we think we would like to test ourselves at home

P5: Erh, I think that testing at home [clears throat] is better, a lot because urh you won't take much time rather than going to the clinic, let's say the clinic is far and taxi fare… maybe you're unemployed so it's better to test at home eerh just to always know but here at home

F: Mmh, okay so maybe how would we feel if we test for HIV at home?

P5: Uhm, testing at home please can I put it like this, it's good, it's bad because erh isn't that you will be counselling after you have found out that maybe you are HIV positive you will need someone who will help you understand this clearly. Let's say I found out again that I am HIV positive, this thing is now a burden for me, how will I counsel myself? I need someone who will help me basically, so it's good to test at home on the other hand its bad

F: Okay, alright then urh is there anyone who will like to add anything else about HIV, that how would they feel if they have to test themselves and why they feel that way?

P2: Erh, testing at home I feel its alright because it takes some people five years of not going to check, because they are scared to check whereas if you check yourself, I feel is alright because…

F: Testing for what sir?

P2: HIV

F: Okay

P2: You are alone at home; you just now prick yourself and check

F: Okay

P2: Yeah

F: Alright then and would you be comfortable to test yourself at home or would you want someone to help you when doing that test?

P2: Oh no, is better if you're alone

F: Alone, okay

Reference 4 - 2.04% Coverage

F: Oh, okay, the rest of us what are we saying with testing at home? Must I test myself or test myself at home but there must be someone to help me still at home? I think I want to hear one by one that's why I keep emphasizing because others are quiet, and we are now at the core of what we are here for. Do you want to test at home or do you want to test…alone or do you want to test at home with someone there to help you or the person helping you must not be present at all?

P3: I would like to say it must be at the clinic

F: The clinic?

P3: Yes, because at home its not great, especially us males its hard because there was a time where we were told to check at the clinic

F: Mmh

P3: We don't want to because us males we make decisions quickly, women are brave

F: Okay sir, so I am hearing you talk about being at the clinic but at the same time you're saying when you're told to go to the clinic you don't want to

P3: Yes, most of the time we don't go as males to go check

F: So, now they are saying they will give you a test that you will do at home, but you said

you don't want to go to the clinic but you also want this test to be done at the clinic?

P3: Ey

F: Isn't they say here is the test we are giving it to you, do it at home so I want to understand

If they give you this test, here it is, we are talking about HIV, is it something that you

would like to do it at home by yourself or would you like to have someone that will help you and be present and do it or be present and you do it yourself but be present so that they will help in case something goes wrong?

P3: Ay, its better if there is someone to help me at home

F: Help you at home and help you do the test?

P3: Yes

F: Okay

P3: Because especially the end results, I will have to tell the one I am married to

F: Mmh

P3: They will then check me and be able to [inaudible segment]

F: Okay

P3: And they will also have knowledge that basically both of us we are like this

F: Oh, so this person must come to test you at home or you want to do it yourself?

Reference 5 - 1.04% Coverage

P2: [clears throat] I prefer that you check at home for someone you know you are comfortable with them, you can talk about everything. Let's say your best friend, someone that will make you understand that no, which decision you must take and eat your pills, not someone who will judge you at the end. Let's say you are now checking, there must be someone or a parent, a parent that will advise you not a parent that will say you go sleeping around and so on, saying negative things

F: Okay, you…from I am hearing from you, you are saying you want to test at home

P2: Yes

F: Be helped by someone you trust

P3: Yes, that you're comfortable with, that you can tell everything to like a friend

F: Oh, like a friend, if it's a nurse?

P2: Even a nurse, if they can come to your home it won't be a problem. Let's say maybe your neighbor is a nurse then you can call them

F: Oh, okay you're against going to the clinic to test for HIV?

P2: You can also go to the clinic, but that thing of checking at home is also alright, both of them are alright

Reference 6 - 1.13% Coverage

: Okay, excuse me, oh okay alright then... then urh what about pregnancy? I am expecting women to…what would we want to…how would you feel if you have to test pregnancy at home?

P5: Testing at home is alright because if you test yourself at home maybe they will say there are some things that you can't like medication you must not take, you will the know that you're pregnant and not wait to go to the clinic

F: Okay

P5: I think testing at home it's alright a lot

F: P4, what are your thoughts on testing for HIV at home…pregnancy at home? And why maybe do you feel that way

P4: Erh I would do it myself at home because of time, and at the clinic there are long lines, you can do it yourself and then when you know yourself and then go to the clinic

F: Oh okay, alright then, there is also a …so you wouldn't want anyone to help you? you wouldn't want a healthcare provider to maybe help you when doing the pregnancy test?

P4: No

F: [giggles] why is it easy to test for pregnancy?

P4: It's just easy, there is nothing to it, you don't need anyone to help you

F: Mmh, maybe is it because we have used it before?

P4: Maybe that's why, yes

Reference 7 - 4.05% Coverage

P2: Oh, diabetes I can say that yeah you have to check because you have to know your sugar levels. Don't say that maybe you are lazy to check at home and find that it's all over your body, you will then have a problem that's why I am saying yeah, it's right to check at home, we can also check it at home, just always check it

F: Mmh, oh, so you need quick results?

P2: Yeah

F: Oh okay, what about the rest of us, how do we feel?

P5: I agree with P2 urh diabetes is a disease that will is very difficult so I think it's really alright that you constantly check at home the situation you are in, is it decreasing, increasing, everything. I think its important to check at home

F: Oh, okay, is there anyone who would like to say something different about testing for diabetes at home and the reasons? Or do we all agree with was said?

All: We all agree

P1: I think that urh maybe if you constantly…you see if you will test yourself at home I'm saying no, when you test at home isn't you will need things to check it, I think it will be more expensive

F: It can be expensive

P1: Mmh, because in this month you will need a kit to check it and next month you will need one again, I think it would be expensive

F: Okay, I am hearing your point on being expensive, we will get to it while we carry on, for now maybe we want to know just about testing, for you to test yourself and how you feel, not that I am not hearing it, I hear it but we will talk about it again but for now we want to know if it could happen that you are able to test yourself at home, do you think it's alright to be able to test for diabetes at home? And do it yourself or to be helped by someone?

P1: Erh I think it's alright, and I think that you have to be helped by someone

F: There must be someone who will help you?

P1: To help you

F: Okay, but at home or help you at the clinic?

P1: Both places

F: Both places, okay then now, BP, the same question how do you feel with doing it yourself urh and why do you think its better to do this thing at home? To be able to test for BP at home?

P4: With BP, you can do it at home but there must be someone to help you because you have to put your hand straight, you can't do it yourself

F: Okay

P4: Yes

F: Okay, and the rest of us? We have chosen BP

P5: I second P4, you need a helper

F: For BP?

P5: For BP

F: Okay, alright urh so maybe over there we spoke about Cancer, I can see from the two that we said are important for us to be able to test ourselves, HIV and diabetes is there, we had mentioned Cancer and said its important to be able to test ourselves, how would you feel? Let's say you are testing yourself, would you want to test yourself at home, would you want someone to help you or would you want to do it yourself? Do you remember we said there are many cancer's so maybe you can talk about the one that you think you can test yourself at home and how would you feel if you do it yourself. Must there be a person who will help or they must be there and sit next to you while you do yourself or they must be there and help?

P2: P2

P4: P4

F: Okay, P4

P4: With cancer its not easy to do it yourself

F: Mmh

P4: 1, there must be a…maybe sometimes they do a biopsy, you can't do it yourself there must be someone to help you with that

F: Yes, mam okay the rest of us?

P2: Uh, cancer yeah you need a helper because it's a very complex disease

F: Mmh

P2: You need a helper to check your blood, blood and everything else [clears throat]

F: Okay

P2: Yes

F: What do the rest of us say about cancer? We said wanted to test for it at home now I hear P4 and P2 say as much as we want to test for it at home but we need [phone rings] but we need someone there to help us, what do the rest of us say? P2, P1, P3, P5

P1: I think that P2 and P4 are right, because maybe if you get a …maybe find that you have cancer maybe cancer of the brain, you will end up freezing from being shocked by that therefore not working

F: Okay, alright we spoke about an app do you remember? Isn't this package that we are dealing with comes with an app?

P5? Yeah

Reference 8 - 0.76% Coverage

P1: Erh I …P1 erh I am saying for me its HIV urh when speaking for myself because I don't have urh I won't say I don't care with the other diseases but I think HIV results are more important for you to keep to yourself

F: Keep to yourself, oh okay but would you want for those results when you have received them to put them in the app?

P1: Yeah

F: You won't have a problem?
P1: I wont

F: Alright, mmmh okay from the diseases that spoke about, we won't have a problem with using the app? Diabetes, HIV and BP? Pregnancy I think you took it out because you said it clearly that you don't have a problem with pregnancy

P2: Oh no, there wouldn't be a problem

F: Mmh

P2: As long as the app says if your blood sugar has risen a bit [mumbles]

F: Okay

P2: Yeah

<Files\\SOUTH AFRICA\\FDGs Community members\\DASH FGD 3 Community members Sweet Water Area(South Africa Rural site) 30 May 2023 (PID 496601602,496601309, 4966041400,496601506,496601705). QC> - § 1 reference coded [8.81% Coverage]

Reference 1 - 8.81% Coverage

Okay, no we are still on the right track. Which ones would we prefer to self-test on our own?

P3: Can I first ask?

F1: Yes, you can ask P3.

P3: Can I ask; if can we include pregnancy (test) there maybe?

F1: Yes, that is a very important question. It is a health condition to be pregnant, is it?

P3: Yes. Can you…

F1: What are the participants in the group saying? What are we saying about pregnancy tests? Should I include it? [pause]

P2: Is it a health condition to be pregnant? [laugh]

P3: No, but it is health-related. [laugh] [cross talk]

P2: Oh! Okay. [laugh] [inaudible segment]

F1: Are we remembering anything else since P3 has said? [pause] Okay… err, okay. So, maybe before we choose which (conditions) we would like to self-test on our own, is there a disease that you think you can explain to us why is it important that we want to test for it in our community? Or any health condition you think you might explain why it is important to be self-tested? [pause] because we don't want to assume we know the answers to why you said these health conditions.

P3: P3!

F1: Yes, P3!

P3: I feel like it is important to know your HIV status, because HIV ends up triggering other health conditions in the body and having a weak immune system, and end up being a person who easily gets infected by diseases. So, knowing your HIV status is very important so that even if you are positive, you can be able to take your medication to minimize being infected by various diseases and end up being a person who is easily infected by diseases anyhow.

F1: Okay. Thank you very much P3. P3 has explained why we want to self-test for HIV. Any others?

P2: I was going to say; P3.

F1: P2! [laugh] [cross talk]

P2: I was going to say, you know I usually say as I was complimenting XXX, they normally come, we were busy voting in the sports ground and they were busy checking us, you know, checking us our chests and all of those things. No, in fact, we have to self-test all the diseases; because if there is… if they say I have an enemy, its like I have HIV or diabetes, I don't know about diabetes now diabetes is now an enemy to me. So, in fact all health conditions should be… that is what I am saying. [inaudible segment] I am putting it all under one banner because we need to include it all and we should not be scared to check.

F1: Is there someone else who wants to express their opinion? Yes, P1.

P1: [inaudible segment] I think it is cancer because you can't just go and check it at the clinic, just like me I am very scared.

F1: It is very scary.

P2: That is one thing, I will be able to self-test on my own or I find someone who will test me at home, something like that because it is very scary [inaudible segment]

F1: Okay, thank you P1. P1 has already answered a question we are about to attempt, but there is nothing [laugh] she has helped us, I now have her answer. So, I was still asking, the question I am asking now: "Which health conditions, from these, do we wish to self-test at home? P1 has already said that she would like to self-test cancer, I am giving it 1, and I will hear from the others which ones they would like to self-test on,-which means I am testing myself. We are still going to attempt the other question but for now, the ones we would like to self-test . [pause] Anyone?

P4: P4

F1: P4.

P4: HIV.

F1: HIV, you can like… okay, are we agreeing with HIV?

All: Yes, yes!

F1: We would like to self-test?

All: Yes!

F1: Okay. Is there another?

P2: Cancer, TB!

P5: TB!

F1: TB, P5. P2 was saying?

P2: Cancer and TB.

F1: Okay. [pause] Is there something else?

P2: We are already self-testing for diabetes. [inaudible segment]

F1: People are now self-testing for diabetes at home?

P2: Yes.

F1: Okay.

P2: There is a friend of mine who is checking himself, just like pregnancy it is also self-tested.

F1: Okay!

P2: [inaudible segment]

F1: Okay… [clears throat] another one? We said we are looking for three health conditions. Are we all agreeing or there is a condition we haven't listed from the three you thought maybe we forgot how important a person should self-test? [pause]

P5: BP is also important indeed.

F1: Mhm, P5 is saying…

P5: [whispering] [pause]

F1: You think it is all right? So, we all agree that HIV, cancer, TB, and BP. But we have a tier between TB and BP because we have to get three. What do we say about this?

P3: TB! [pause]

P2: For me, we rather leave out TB. [whispering]

F1: Why should we leave BP and take TB?

P3: BP is a common health condition.

F1: Yes.

P3: TB can make you end up in a hospital, and you will end up feeling like distancing yourself from other people because others would want to isolate themselves because you have a TB that is contagious. Whereas BP, there is nothing much about it.

F1: [giggles] Okay, is there anyone who would like to assist P3 with what she has said or add to what has been said? Do we all agree?

P2: She has said…

F1: Okay. So, we have HIV, Cancer, and TB. The health conditions we would like to self-test on our own. Now, the health conditions we would prefer if we test and have rapid results. It can happen that we have already mentioned them from these three, you can also mention them, but it does not mean we will no longer list them. The conditions we would like to test and have rapid results which means, if you have this (condition), the process of taking blood samples today and having your results the other day, we are not mentioning the ones you will test yourself, but we are saying you will get rapid results. It might happen you don't self-test, but err, you would like rapid results and how it is performed. [pause]

P3: Cancer!

F1: Cancer. Okay, what are others saying? [pause]

P2: TB!

F1: TB. [pause]

P1: HIV.

F1: HIV. [pause] We are working together here, if there is something you think we are forgetting, you can mention it. [pause]

P3: Meningitis!

F1: Meningitis. [pause] [writing on board] We can still mention if there are others. [pause] [writing on board] Is there something else we would like to get rapid results? [pause] If there is none because we mentioned; HIV, Cancer, TB, and meningitis. Is there one we would like to let go of from these three? We can all agree on which terms are we letting them go.

P3: P3!

F1: Yes, P3.

P3: I think it is HIV because there are kits already where you get your results same time.

F1: Mhh. We are able to easily access it?

P3: Yes!

F1: Okay. What are others saying?

P2: Yes, I agree with her, I second her. HIV is something you get now, whether you have it or not.

F1: Okay. So, with rapid testing we are saying; cancer, TB, and meningitis? [pause] [writing on board] Is there something we are leaving behind or anything you are thinking? [pause] [writing on board]

P2: Gout!

F1: [laugh] Yes, what is P2 saying about gout?

P2: I am saying, ey, it does hit hard on people.

F1: Mhhm.

P2: Ey, it's [cross talk] people end up visiting traditional healers because it becomes swollen, you see?

F1: Oh! It normally appears through having swollen feet. [cross talk] Does it normally appears through swollen feet to people?

P2: Mhh, you see one of my friends, his fingers are like… he has numerously consulted traditional healers, but it is found that it is gout.

F1: Mhhm. [pause] Okay. Others? Are we still on the meningitis, cancer, and TB? [pause] [whispering] Okay. [pause] TB, ey. I hear the issue of gout… really if it appears the way it does, it makes one not know [cross talk]. Yes.

P2: The way I am looking at it, a person should know themselves because they might think it is a condition related to tradition, and end up running around only to find its gout.

F1: Okay. Is there someone we know who ended up facing gout condition?

P3: Yes, there is.

F1: There is?

P3: Yes.

F1: Okay. Okay, thank you very much for your answers. We will now proceed forward. P3 once mentioned the issue of pregnancy tests. I wish to ask because we didn't mention this one in the health conditions we mentioned. So, what are we saying about Sexually transmitted infections, what is called STIs? Do we see it as important enough to be tested, is it prevalent within our communities where we would like it to be tested or do we want it to test it at our homes? Or is it already being tested, I need to hear your opinions about it.

P3: Ay, STIs are not common to be tested or it is a health condition people are more commonly with it or they are more exposed to it. You will always find few people who know about STIs, they just do [laugh] but seriously we do not have much information about STIs, no.

F1: What are others saying about that? [pause] Do we know or not whether at the clinic we can test for it?

P2: No, we do test for it at the clinic, and we also get help regarding STIs, I agree with P3 that it is not that important for us to go and test for STIs because in some instances, you do get assistance at the clinic, and also people who are suffering from such health condition they are few, you see? Again, those people put themselves intentionally in danger, you see? [laugh]

F1: Yes.

P2: So, I agree with P3 that no.

<Files\\SOUTH AFRICA\\FDGs Community members\\Dash FGD-02- Community Members Sweet Water (South Africa Rural Area) 23 May 2023 (PID 496601204,01101,496600901,496600809))Transcript.QC> - § 3 references coded [12.46% Coverage]

Reference 1 - 6.54% Coverage

Oh. Okay is there anything that we are forgetting, is there anything else? [Silence] Okay, I here that you are saying that these are the common illnesses in your communities or in the communities that we live in? I would like to know which ones would you want to conduct a quick test for; that could provide you with result in a short period of time? Three which you think you would want to get the result for quickly. So, we will work together to choose just three. And again, we will assist each other to choose another three for which you would like to self-test for;that I can test my self at home. But for now, I would like us to help each other to identify which three. If there is any that you had forgotten you can still say it. Which three would we want to be able to test today and have result for it today? [silence] That which we think is so important to a point that we have to test for it today and get the result for it today.

P2: I 2P, there is this one that has arrive called Monkeypox.

F1: Okay, Monkeypox. We had not written it, I am writing it now.

P2: Maybe before we go into choosing them, what are your thought about sexually related disease the one's we call STI's, any that are related to such. Maybe, do we want them to be tested rapidly?

P2: Yes.

F1: Are they common in our communities?

P1: A lot.

G: Yes.

P3: P3.

F1: Yes P3.

P3: I had also forgotten that one, but that disease affects everyone who did not take care of them self by using protection when having a sexual intercourse.

F1: Okay, so did we forget it, can I add it?

P3: Yes, although I do not know what do others think.

F1: What are other's saying?

G: Write it.

F1: I must write it. okay.

P2: and Shingles.

F1: So, Shingles.

P3: [Whispers] What is that?

P2: [Laughs].

G: [Laughs].

F1: Can you explain for us what is Shingles? You can just explain what you know about it.

P2: I just know the body gets itchy, it is something very painful, and looks like you have a belt.

F1: Oh, okay.

P5: Such as this thing, what is this called? Hives.

P2: Yes, like you have hives, the entire body get itchy.

P5: You get itchy all over the body.

F1: Okay, what do you think about pregnancy. We did not write it here, maybe that was because I had asked about diseases, maybe just to clarify that even just conditions. What do you think about pregnancy, we have women in this room, is it something common, would we like to test for it, is it something that we think our community would benefit from testing?

P2: Yes, I think that that they would benefit…

F1: Uh-hum.

P2: I as P2, I do think that the girls, especially the youth should go test, or maybe have a way in which they can protect themselves so that they do not end up in a situation where they have fallen pregnant.

F1: Um, okay. Okay as I had already said we are now going to look at this list, see and try to find out which top three do we want to be tested for and get quick result for. Let's help each other now. What do you think of BP, are we including it in the top three that we are trying to get? [Silence] Or is it not important maybe? I do not know but I want to hear from you. We are looking for the three that we want to be tested rapidly in our communities.

P3: P3.

F1: Yes, P3.

P3: It is cancer.

F1: Can you explain why cancer?

P3: It kills if you are not aware that you have it, even if you know that you have it. When you are being treated for, it kills you.

F1: Someone else.

P5: It is TB, HIV, can I say all three, the ones that I think could fit there?

F1: Yes, you can say them. You said it was TB…

P5: Uh, HIV

F1: HIV.

P5: And this one, BP.

F1: And BP. Do you want to explain to us, is there any you want to use to explain?

P5: No, these are the ones I know to be common. They are just common.

F1: Um, okay they are common. Okay, anyone else, maybe is there anyone who want to also state which one's do they think-

P2: Uh, P2 I do not agree with BP, because it is not a big thing, what I think could be is Corona (Coronavirus).

F1: Okay so you think that it would be Corona?

P2: Yes, Corona.

F1: Okay, what do others think? Maybe lets start here, what do others think regarding-since we are trying to get three diseases that we can come out with. P1 what are your thought? On these three diseases, can we put BP?

P1: Yes, we can put it.

F1: Okay, BP has gotten two. Father, what do you think of BP, are we putting it or not?

P1: It is important.

P3: Let's put it, it commonly affects elderly people.

P1: What about me since, it affects me as well?

G: [laughs]

P1: I also take pills for BP.

F1: Yes.

P3: But, eh…

P1: There are many diseases.

P3: There are many and they are different.

F1: Okay, is there anyone else who want to add any diseases before we vote? or do you maybe agree with these ones?

P1: Yes, the ones that have been ticked.

F1: We have one,two, three, four,five diseases and we are trying to understand which ones are among the three. There is cancer, HIV, TB, BP and COVID. Which ones do you agree with for rapid testing?

P1: It is TB and HIV.

P5: Me too, it is TB and HIV.

P2: Uh, the same.

F1: What are we saying about COVID since we mentioned it?

P5: As the sister had stated,there is an area where it is still killing people.

F1: Yes.

P2: Yes.

P5: Let's include it.

P2: The worst part is that COVID is worse than BP. With BP you can get medication and get treated. With Corona you get injected, but still die. There are a lot of people who got injected with that injection but died. So, with BP you get medication but you don't die, it helps you and heals you.

F1: Okay, what are we saying between-please correct me if you feel I am not understanding what you are saying. We said it was TB and HIV, it seems as if that is without doubt.

G: Yes.

F1: So now, what we are trying to decide on is COVID and BP.

P1: No, it's corona.

F1: What are we agreeing on?

P5: It's corona.

F1: So, the disease that we want to be tested on rapidly is TB, do help me.

G: HIV

F1: Yes.

G: Corona. [Door opening] [Door closing]

F1: Okay, we are now going to look for the one's that I wish to self-test myself for. What diseases do you think if services were brought that you see this one, I wish I could test by myself-and just take the thing and…

P3:P3.

F1: Yes.

P3: It's cancer.

F1: Okay. Others? Can we please just mention them as I'm writing. Others, which other one's the father has said that it's cancer, P3 said cancer.

P5: P5

F1: Yes, P5.

P5: It's STI's.

F1: It's STI's. You would like to test it by yourself?

P5: Yes.

Reference 2 - 3.37% Coverage

P3: If you know about it, you cannot pass it on to another, it is just important that there be a way to self-test, to self-test alone and then you go seek for help where you can get it.

F1: So, do we agree that this one you would test by yourself, what else? We are looking at what else do we wish to test by yourself?

P2: [Coughs] I have a question. From the ones we have noted such as HIV, are we able to follow up on the ones that you need to teston?

F1: Yes, you can. If you see one that we have put here already but if you believe that if could help me the most to be able to self-test it you can mention it. So, which one, is there any that you are thinking of P2?

P2: No, it is just a question.

F1: Is there anyone else, we need three diseases that we would like to self-tesfor,;which ones would you want to self-test for? [Silence]

P2: I P2 I think it is BP.

F1: Okay, you would like to test yourself for it?

P2: Yes.

F1: Is there a reason?

P2: I do not see the purpose of going to the clinic because you would just test yourself, get your result and then you can be able to go get your medication at the clinic instead of going to stand in multiple queues.

F1: Um.

P2: Yes.

F1: So, do you feel that you would need to test your self-first,

P2: And than just go there…

F1: And only go there to say, this is what I have discovered, please help me with such

P2: Yes, yes.

F1: Okay. Any one else, we have cancer it has only one, is there any one who would like to test themselve for cancer?

P2: Yes.

F1: Is there a reason that you would like to give for that, any one can help them as I have said that your opinions are important, anyone? We are all having this discussion together. Is there a reason why some one would want to self- test for cancer?

P3: P3.

F1: P3.

P3: What I see as important as I've heard such good news, self-testing and seeking help is important. It is cancer, it is cancer.

F1: So, you also agree with P2?

P3: Yes, yes.

F1: Father, is there anything else that you wish to state regarding this?

P3: Because it is nice when such opportunities have come to now look at yourself and then start seeking for information on how you can be assisted.

F1: Okay, we have, uh, we said it was STI's, BP and Cancer. Is there anything else or do we all agree with what has been said?

P5: Yes, indeed, BP. Yes, it is alright.

P2: We agree with what have been said.

F1: Let us just have a look at them. We have all these diseases such as corona. Uh, and maybe asking for myself what are we saying about a pregnancy test, what are your views about it? Don't you want it to be self-tested or maybe is it there, what's the story with it?

P3: I wouldn't have an opinion on that one.

G: [Laughs].

F1: So, you don't have any opinion P3 [Laughing].

P5: No, with pregnancy, because you can get this, these things for testing are accessible in the clinics already-

F1: Oh, it's already accessible?

P5: It is not too important.

P5: Even in the stores, you can buy it and go test yourself, to help yourself.

F1: Oh, it is accessible in the shops.

P5: Yes.

F1: Oh okay, is there anyone who still wants to say something on this?

P2: On pregnancy?

F1: No, just on this phase where I had asked about what we want to self-test for, what do we want to test ourselves for. Is there anything else we have left?

P2: No, I think [Phone rings].

Reference 3 - 2.55% Coverage

F1: From all of these which one would you like to able to test for at home?

P3: That I have never tested for?

F2: No, even if you have tested for it before but whatever that you would like to test for at home from all these things I have shown here. [Silence] Here are different testing kits so you can see which one would you like to test at home from all these. Is it testing for diabetes, testing for HIV, testing for Malaria, testing for COVID , diabetes, or pregnancy, which one would you love to-

P3: For me, all of these.

F2: You would like to self-test for all of these. Others?

P2: I P2 I think it would be testing for pregnancy as I am a female.

F2: You would like to test for pregnancy

P2; Yes, at home.

F2: Just because you are a female, is that the only reason you have?

P2: Yes.

F2: You would like that just because you are a female?

P2: Yes.

F2: Okay, since-

P2: And then it is diabetes

F2: Okay P2 is saying it is pregnancy, the father is saying it's HIV, other?

P3: No, I said I have tested for it.

F2: I am asking which other one would you like to test for at home by yourself?

P3: Malaria.

P1: Checking for HIV.[Silence] [Paper shuffling]

F2: Okay. Are those the only things you would like to test for?

P5: Uh-huh.

F2: Why would you like to test for HIV, mother?

P1: To know about myself, what the health is the condition of my blood.

F2: Uh-huh, so P2 why would you want to test except for that you are a woman? That which you want to check for-

P2: Okay, it is because going to the clinics or going to buy testing tools is work, or it is extra work. And once you have tested and done that you could mistakenly live this at home or somewhere, and then people in the house see it when you had not wanted them to see such.

F2: Okay, father what about Malaria?

P3: We have, I have a lot of mosquitoes there, especially in summer.

F2: Yes.

P2: Malaria kills.

F2: X would you please open for me that thing, so I can show them the actual app. So that they can talk about something they can see and touch because we have to know how they would feel about using something like this. When you actually do the test would you like to test yourself or maybe would you like to be tested by someone from a health facility? You are testing now you have made a choice whether you want to test for pregnancy or HIV or Malaria. Do you want to self-test alone in your home with your app, or would you like to have an app but also have someone from the clinic or just someone else to help you continue with testing yourself?

P3: P3.

<Files\\SOUTH AFRICA\\FDGs Community members\\Dash FGD-04- Community Members Sweet Waters (South African Rural site) 13 July 2023(PID 496606305, 496606203, 496606100, 496606404, 496606009) Transcript. QC (1)> - § 2 references coded [5.53% Coverage]

Reference 1 - 4.15% Coverage

F1: So, right now, I would like to know, Eh, sorry [Whispering]. So now from this, from this top three. Can we please arrange them according to the ones we would like to self-test for, when I speak of self-testing I am referring to being able to test ourselves. We are now choosing from these, right now they are just placed randomly, but I now want us to arrange them as this is our top1, our top 2, top 3 that we would want to self-test for. [Silence]

P3: Cancer.

F1: P3 what number is cancer? Okay, number 1.

P5: HIV.

F1: Is HIV number 2?

P5: Yes.

F1: P2.

P2: HIV.

F1: HIV, so do we all agree that Cancer is number one?

P?: Yes.

F1: We are saying that HIV is number 2.

G: Yes, Yes.

F1: Meaning BP will be the last one. Oh, they will continue to be like this oh let me put this here. Okay, I see that you have mentioned illnesses like cancer, SIT, TB, mumps, and so on, eh I would like to know what are your thought about pregnancy.

P5: It also goes in.

F1: It comes in?

P5: Yes.

F1: Anyone else? What do others think about pregnancy because we should not just take an answer from one person? We can just talk and be free.

G: [laughs].

F1: You can place even your bag on the table, so you can talk freely, you see.

G: [laughs].

P3: Our youth needs to abstain from sex.

F1: Yes, oh so you don’t think that pregnancy should be included here?

P3: It’s not an illness.

F1: It’s not an illness?

P1: I also agree with that, it’s not an illness.

P3: It is not an illness.

F1: Though it is not an illness, in your perspective is it important for women to be able to self-test quickly for it?

P3: If they prevent meeting up it won't be there.

P1: Yes, that's why.

P3: You don’t just wake up and find it there. You won’t be pregnant.

F1: P4 now?

P4: It is important to test because sometimes it is done to quickly protect the child if in case their parent is not alright.

F1: Yes, so it you P4, P5, you two agree with each other. P2, what are you saying, is it important for people to be brought self-test so that they would be able to test for pregnancy quickly?

P2: Eh, it's important.

F1: Eh, will write up here, some say it is important, and some say it is not. So, now I want us to talk about each test. Right now, will talk about cancer one, so what kind of people do you think this cancer test will be suitable for? What type of people would it be suitable for? Who would it be suitable for to test for cancer?

P2: P2

F1: Yes.

P2: Those who smoke.

F1: Those who smoke, okay. P3?

P3: I would say everyone because women have breast cancer, there is cancer for genital area. There is throat cancer, skin cancer you see, you can’t not say it is because someone smokes, a mother who doesn’t smokes has breast cancer.

F1: Eh, okay, thank you P3. Is there anyone else, who can tell us what kind of people is the test suitable for, the cancer test? Ah P5.

P5: I would also say it, everyone, because you get cancer even if you do not smoke, or do anything, you get cancer.

F1: Okay, is there anyone with something different from what they have stated, such as smoking and that everyone should test.

P4: I P4 also agree with that everyone, because it does not choose.

Reference 2 - 1.38% Coverage

P1: Even in the house you can do it, it is not prescribed that have to go to the clinic. What could be easy is for these testing to be available, and be accessible. Isn't it that condoms can be found anywhere, they too should be accessible to people so that people can take it for themselves and go and self-test before coming to the clinic to get help.

F1: Uh-hm, since you are saying everyone should test for HIV, what would be the reasons that would make these people test for HIV? What do you think should be the result?

P1: Firstly, women are breastfeeding, let us say that, so if it happens that you have given birth to a baby, and then you become intimate with someone, you didn't have it whereas they have HIV. In that duration you are still breastfeeding, who will that affect at the end, it will affect the child. So, it is better to have it with you, so if you know that you are sexually active but you do not use protection or you do not eat these things that are taken to protect HIV, you can keep testing yourself in order to always protect your baby.

<Files\\SOUTH AFRICA\\FDGs Community members\\DASH FGD-05-Community Members Sweet Water Area (South Africa Rural Site) 14 July 2023 (PID 496606706, 496606808, 496606902, 496607003, 496607106) QC> - § 1 reference coded [13.03% Coverage]

Reference 1 - 13.03% Coverage

F1: Okay so we are going to rate now and all of us as a group should rate. We should have top three diseases which we think would be great for them to have rapid diagnostic test that can be used by people So we have to rate our tope three here. A person will raise a hand to rate a disease that you vote for but when we vote, we have our top three in the end of those that we would all like to have rapid diagnostic test where you can get your results fast when you are testing. Okay, we start voting.

P2: P2.

F1: P2.

P2: Cancer.

F1: Cancer.

P4: Err P4, HIV.

F1: HIV.

P5: P5, TB.

F1: TB, if you agree that it is TB, you can also mention it.

P?4?: [Inaudible segment].

P1: P1, cancer.

F1: Cancer.

P5: Diabetes. [Cough].

P3: P3, cancer.

F1: Cancer.

P5: P5, I also agree that it is cancer.

F1: Cancer.

P1: P1, HIV.

F1: HIV.

P4: P4, HIV.

F1: P2

P2: Heart diseases.

F1: Heart diseases, okay anyone else?

P4: P4, heart disease again.

F1: Heart disease, we can continue.

P4: P4, cancer.

P5: P5, TB again.

F1: TB.

P1: P1, HIV.

F1: Alright, thank you. So, the first one is cancer, HIV is second hmm.

P?1?: It is a draw between TB and a heart attack.

F1: TB and heart attack. Ok between TB and heart attack?

P4: Heart attack.

F1: What are we saying?

P5: P5, it is TB.

F1: TB, what is another one saying? Is it heart attack or TB?

P4: Heart attack.

F1: What are you saying?

P1: TB, two.

P2: Heart disease, two.

P3: TB.

F1: TB three which means we are going with TB. Alright [cough] so on these three diseases that we have here, which, this is our top three so now want top three from self-testing but we will take it here. Which one do we think people should do it themselves when they are self testing hich one is number one so we will do it in sequence like one two three depending on which one do we think people would be more comfortable to test themselves another one where people would be comfortable and the last one so we also have to vote here too. Okay.

P4: The first one is HIV.

F1: You think it is HIV.

P5: P5, HIV.

F1: HIV, we do agree that HIV is number one?

P5: Yes.

F1: Which is the second one that people can be able to test themselves.

P4: P4, it is TB.

F1: TB.

P1: P1, TB.

P3: P3, cancer.

F1: Cancer?

P3: Mm hmm.

F1: Number one.

P2: P2, TB.

F1: Three.

P5: P5, TB.

F1: TB is number two and the last one is HIV so maybe what are other_

?P1?: Sorry

F1: Yes.

P2: What is the last one?

F1: It is cancer, thank you. Okay uhm so now we will look at these three diseases that you have given us and we will start with HIV. Do you think these diseases, who should test for HIV? Who should get tested for HIV?

P4: Youth.

You have to say P who.

P4: P4, youth.

F1: You think that it is the youth.

P4: Yes.

F1: Okay, what do others think? [cough]

P5: It should be everyone.

F1: Everyone, who should get tested according to others?

P3: I also agree that it should be everyone.

F1: Everyone, others?

P1: P1, everyone should get tested.

F1: Everyone okay youth it's the youth and others are saying everyone. Okay where do you think they should get tested for HIV if people are testing?

P2: [Inaudible segment] err I think that they should get tested where they are comfortable to do it since nowadays, there are test to, self-kits to test yourself at home. You can test yourself there but if you are not comfortable, maybe you can get tested at the clinic by another person and tell you your results.

F1: Hmm okay, what do others think people should get tested for HIV?

P5: P5, I also think that it is better if you test yourself at home, buy test kits from the pharmacies and wherever you feel comfortable or at the clinic.

F1: Yes.

P3: Err p3, I think that it is better to get tested at the clinic because I think that at a clinic you get someone to give you counselling err since it is very hard for us as people to understanding this HIV

thing. Someone may think that it is the end of the world after hearing th he/she is HIV positive but will live So that's why I think a clinic is alright because you might a person who will convince you and be okay.

F1: Okay, p4?

P4: P4, I also think that it is good idea to get tested at the clinic because when you test at the clinic, they can know your blood type because when you get tested for HIV, they give you more information and you may find that you are blood o which is maybe a stronger blood since they have lots of information at clinic [inaudible segment].

F1: So that they can also tell you, is there anyone who wants to add anything other than a home and a clinic? Okay according to your thinking, what should be a reason for testing HIV? What should make someone to decide on testing HIV or want to get tested?

P5: P5

F1: Yes

P5: Err the reason for someone to test for HIV is to know if he/she has it or not and get medication before he/she gets sick.

F1: Why do people get tested? If you agree with p5 you can say so but if there is someone who has something different, he/she can simply say he/she has a different point. [Laugh] okay its fine, so how should HIV people link to care?

P5: What kind of help?

F1: Help that follows after testing. Help that follows after testing, let's say you tested positive so how do you think people should be linked to care? Let's say you tested yourself so how can be linked to care? How would you like to link to care after self testing, how would you like to move forward and get linked to care?

P3: Oh p3.

F1: Yes p3.

P3: Err I would say if you find out that you are sick, you should go to the clinic and get medication so that we can be well.

F1: Do you agree? I see you doing this with your heads [laugh]. Okay we are now moving to TB, who should get tested for TB according to your thinking?

P5: P5, I think everyone should get tested for TB?

F1: Everyone, another one?

P3: P3.

P4: P4.

F1: Can we please start with p3.

P3, err I think that anyone who is coughing like p2 [laugh]. Anyone who suspects that there is more since he/she keeps coughing.

F1: [Laugh] okay p4, you raised your hand.

P4: Yeah, I think that everyone should get tested because it is an airborne disease like Covid so everyone.

F1: Everyone because it is an airborne disease.

Group: Yes.

F1: Okay err do you have a reason for saying everyone should get tested?

P2: Err I do have a reason?

F1: What is your reason?

P2: Err you can get TB as new-born because it is airborne and it is not easy to protect yourself so it is important for everyone to get tested occasionally.

F1: Okay.

P2: Because sometimes it is a disease that hides and comes out when it is hard to stop it

F1: So, when a person test and find out that they have TB, how can they be linked to care since we said that in terms of HIV, they have to go to the clinic so what do we think when it comes to TB? Is there any other different way or what do you think?

P2: I don't see any other different way beside going to the clinic [mumbles] yeah.

F1: Is that p2?

P2: Yes, it is p22.

F1: Okay, p4.

P4: I agree with p2.

F1: You agree with p2, okay. We are now moving to cancer [exclaims], we are now moving to cancer now. What kind of people should get tested for cancer according to your thinking?

P2: Everyone should get tested for cancer.

F1: Reason?

P2: Because cancer has stages and on others it is said, what, is it the one which is hereditary, isn't it? They say it is hereditary.

F1: Mm hmm.

P2: Yes, now you don't know and maybe by the time you find out what is making you sick if you are sick and don't know what's wrong then it is late to err be treated.

F1: To be treated.

P2: Yes.

F1: Okay uhm alright, do others agree with p2 or we have other opinions?

P4: We agree with p2.

F1: You agree with p2. Okay those who have cancer, how can they be linked to care?

P4: P4, they should go to the clinic.

F1: To the clinic, so everyone should go to the clinic [cough] and not go to a doctor, a private doctor?

P3: [Exclaims]

F1: [Laughs] okay why are you exclaiming p3?

P5: They can go to the private doctor but we are not encouraging that because there's no money, people are unemployed so you so you should have money to go to the private doctor.

Files\\DASH IDI, Community Stakeholder, Sweetwater (South Africa Rural Site) 13 July 2023 (2) - § 1 reference coded [2.63% Coverage]

Reference 1 - 2.63% Coverage

I: Eh, perhaps what information could community members be able to gather that can be put in the dashboard so that we can better understand their needs?
P: Eh, even though it's not that easy in terms of people being able to gather information by themselves.
I: Yes.
P: Because let me make an example about this modern technology of giving a person an app where they test themselves at. It's their thing and they can't tell other people. And no one knows that they have this app of testing yourself and sending the results. A person knows themselves.
I: Yes.
P: Unless the facility asks the community leaders to gather people so that they can come and talk to them.
I: Yes.
P: Besides pointing out that they have helped certain individuals or not, but to just generally talk to the people and give them full information and then it will be up to the people to decide what they do with it.
I: Oh, decides for themselves.
P: Mm.
I: Eh, so do you think community members would be able to understand something written such as this?
P: As you can see, you're doing this eh, I take it as a presentation.
I: Yes.
P: As you've gathered people before doing the handout you will first talk to them, and they will read with understanding.
I: Mm.
P: Yeah, what's important is to make a person understand.

Files\\DASH IDI, Community Stakeholder, Traditional healer, Sweetwater (South Africa site) 04 July 2023 PID 496602907 (2)-QC - § 2 references coded [2.98% Coverage]

Reference 1 - 1.81% Coverage

I: [giggles] Okay, umm, so what do you think about the patient using an App like this one I have shown you, to be guided through self-testing and next steps, such as home care or getting advice from a healthcare provider via the App?
P: No, [clear throat] I think it is very good to get advices about healthcare because even after you have tested at home, it would be better if they intervene and come to you and speak with you to give you hope because in some instance, you can be tested at home by your kids, but you have some doubts and being terrified and you might even lose hope about life, but if they intervene and explain to you that since it is like this, you must have hope that life goes on, the only thing you should do is that you should take care of yourself and proceed with the way in which you were told, you will live a longer life.

Reference 2 - 1.18% Coverage

I: Yes. So, they can have a communication on their own with the healthcare provider on their phone?
P: Yes.
I: And they can be all right, that it is only them and the healthcare provider who knows, not everyone.
P: But it would be important for someone at home to know their secret.
I: At least one?
P: Yes, at least one who knows, like HIV. With HIV, you have a partner who accompany you to the

clinic who will be responsible to remind you if it's time that now it is the time, etc.
I: Mhm.
P: Yes.
I: That one person you trust.
P: Yes, the one you trust.

Files\\DASH IDI, Community Stakeholder, Ward Councilor, Sweetwater (South Africa Rural Site)14 July 2023 PID 49-66-059-0-6 -QC - § 1 reference coded [1.10% Coverage]

Reference 1 - 1.10% Coverage

I: Okay err what do you think about community members using an App to certain instructions on App? Maybe like do this and this.
P: It will help a lot because once you have tested and find out that you are positive, you will get instructions telling you to go to the nearest clinic…
