# Supplementary material for: Disease priorities and rapid diagnostics testing preferences among community members in KwaZulu-Natal, South Africa: a formative qualitative study
Source: BMJ Open. 2025 Nov 19;15(11):e104997. doi: 10.1136/bmjopen-2025-104997 (PMC12636905; doi:10.1136/bmjopen-2025-104997)
Supplement: Supplementary file 3 [file bmjopen-2025-104997.reviewer_comments.pdf]

PEER REVIEW HISTORY

BMJ Open publishes all reviews undertaken for accepted manuscripts. Reviewers are asked to complete a checklist review form and are provided with free text boxes to elaborate on their assessment. These free text comments are reproduced below.

ARTICLE DETAILS

Title (Provisional)

Disease priorities and rapid diagnostics testing preferences among community members in Kwa-Zulu Natal, South Africa: A formative qualitative study

Authors

Madonsela, Thandanani; Naiken, Lenika; Lauff, Amber; MWAMBA, CHANDA PRUDENCE; Sharma, Anjali; Maritim, Patricia; Tshazi , Ayanda; Bemer, Meagan; Morton, Jennifer F.; van Heerden, Alastair; Humphries, Hilton; Drain, Paul K

VERSION 1 - REVIEW

**Reviewer**	**1**
**Name**	**Wiens, Kirsten E.**
**Affiliation**	**Temple University**
**Date**	**28-Jul-2025**
**COI**	**None**

In this manuscript, the authors aim to understand the perspectives of community members in uMsunduzi, KwaZulu-Natal, South Africa regarding: 1) which diseases should be the highest priority for improved diagnosis and treatment, and 2) which diseases they would support for rapid diagnostic tests (RDTs), including home use. This is an important study with policy-relevant findings. However, before publication, the manuscript requires additional detail on the qualitative data analysis methods as well as a clearer description of the findings in the results section.

Major Comments:

1) The methods section requires further detail on how the study was conducted:

• Page 7, lines 29-30: Please clarify what is meant by "community key stakeholder" and describe how the purposeful sampling was conducted.

• Page 7, lines 44-46: Where were community members recruited (e.g., door-to-door visits, community centers, or hospitals)?

• Page 7, lines 51-53: Was there any concern that having focus group discussions mixed across age and gender might prevent individuals from being as honest or open as they might be in demographically homogeneous groups?

• Please detail how people were selected for focus group discussions (FGDs) versus individual interviews (IDIs). Did any participants take part in both?

• Page 8, lines 14-19: Did all three reviewers code the transcripts independently and then compare results? How were conflicts resolved? Were any other procedures used to minimize bias and ensure consistency (e.g., maintaining personal and analytical notebooks throughout the process)?

• What process was used to create the qualitative codes? Were the codes developed deductively (pre-defined based on existing theories) or inductively (while reviewing the transcripts)? Please detail this process and, ideally, provide the codes used in a supplement.

2) How were the priority diseases listed in the results identified? Are these diseases that were mentioned at any time by any participant, or were they repeatedly mentioned? Please clarify the selection criteria and thematic analysis process.

3) The transcript segments included in the results are not particularly helpful. I recommend including only key quotes from participants that illustrate the main themes that emerged from the analysis; full interview segments could be included in a supplement. For example, this approach was executed well on page 11, lines 27-47.

Minor Comments:

• Introduction: It would be helpful to add details regarding which RDTs for high-burden diseases are currently available but require resources for rollout, and which are still in development.

• Page 7, lines 10-11: Please add appropriate references for the DASH study.

• Methods section: It would be helpful to include in parentheses the initials of the co-authors who contributed to the different components of data collection and analysis.

• Table 1: Percentages are missing for the study population breakdown by age group.

• Results section: It would be helpful to know how the demographics of the participants compare to the broader population. For example, is it expected that 66.7% are unemployed (and are these mostly caregivers?)?

Reviewer 2

Name Scott, Godfred Yawson

Affiliation Kwame Nkrumah University of Science and Technology College of Health Sciences, Department of Medical Diagnostics

Date 29-Jul-2025

COI **None**

Overall, this study is well-written and designed. It addresses an important and timely issue concerning community disease priorities and rapid diagnostic testing preferences in KwaZulu-Natal, South Africa.

However, I recommend the following improvements before publication:

1. Writing Style and Language:

The manuscript would benefit from a thorough review of grammar, punctuation, and sentence structure to enhance readability and flow.

2. References:

Please carefully review and verify all references. Additionally, the reference list includes works as far back as 2002. While some foundational references are acceptable, please consider updating citations with more recent literature to reflect the current state of research and policy, especially in this context.

VERSION 1 - AUTHOR RESPONSE

RESPONSES TO REVIEW 1'S COMMENTS

Reviewer 1 comment: 1) The methods section requires further detail on how the study was conducted: Page 7, lines 29-30: Please clarify what is meant by "community key stakeholder" and describe how the purposeful sampling was conducted.

Author response: *We defined community key stakeholders as individuals with a leadership or public service role, such as local leaders, ward councillors, traditional leaders and traditional healers. This has been clarified in the manuscript pages 6-7, line135-145*

Reviewer 1 comment: Page 7, lines 44-46: Where were community members recruited (e.g., door-to-door visits, community centers, or hospitals)?

Author response: *The stakeholders who agreed to participate were enrolled in the study. We conducted community drivers in busy communal areas including taxi ranks and shopping areas to recruit community members. This has been clarified in the manuscript page 6 - 7, lines 135 – 145.*

Reviewer 1 comment: Page 7, lines 51-53: Was there any concern that having focus group discussions mixed across age and gender might prevent individuals from being as honest or open as they might be in demographically homogeneous groups?

Author response: *While there may be a concern that conducting FGDs that are mixed across age and gender may limit openness for some participants. To elicit diverse perspectives and enrich the discussions, we intentionally included demographically diverse participants, which also allowed participants to reflect on others' experiences. As a mitigating factor, the qualitative interviewers are trained and experienced in facilitating group discussions with demographically diverse groups.*

Reviewer 1 comment: Please detail how people were selected for focus group discussions (FGDs) versus individual interviews (IDIs). Did any participants take part in both?

Author response: *Qualitative data were collected over time and in phases, enabling the study team to track participant profiles and purposefully assign participants to either IDI or FGD. No participant participated in both FGD and IDI, ensuring diverse participant perspectives, minimising repetition, and preventing participation fatigue. Participants were invited to FGDs based on their availability for a scheduled FGD and demographic profile to ensure diversity in the group.*

For IDIs, participants' demographics were systematically tracked by the study team to ensure participants of different ages and genders were interviewed within the protocol-defined sample size for community members IDI.

The community stakeholders were only invited to participate in IDIs due to scheduling constraints.

Reviewer 1 comment: Page 8, lines 14-19: Did all three reviewers code the transcripts independently and then compare results? How were conflicts resolved? Were any other procedures used to minimize bias and ensure consistency (e.g., maintaining personal and analytical notebooks throughout the process)?

Author response: *Two of the researchers jointly coded the transcripts to keep the codes consistent, and the third reviewed the codes and excerpts. Discrepancies were discussed between the researchers until a consensus was reached. Through iterative reading of the textual data, additional codes and emerging themes were identified, and the codebook was amended as necessary. Excerpts regarding disease priorities and preferences for RDTs were extracted into Microsoft Word reporting. We used thematic content analysis to categorize identified codes into broader themes.*

This has been clarified and integrated in the manuscript on page 7-8, lines 169-177

Reviewer 1 comment: What process was used to create the qualitative codes? Were the codes developed deductively (pre-defined based on existing theories) or inductively (while reviewing the transcripts)? Please detail this process and, ideally, provide the codes used in a supplement.

Author response: *The preliminary codebook was developed deductively based on concepts and frameworks related to the study objectives. Revised in the manuscript, page 8, line 205*

The codes used are provided in a Supplement 1.

Reviewer 1 comment: 2) How were the priority diseases listed in the results identified? Are these diseases that were mentioned at any time by any participant, or were they repeatedly mentioned? Please clarify the selection criteria and thematic analysis process.

Author response: *The priority diseases and preferences for RDTs were identified inductively from participants' narratives during FGD and IDs. Diseases that were either repeatedly mentioned across participants or where in the FGD a consensus was reached, were categorised as priority diseases. No disease categories were predetermined in the codebook; instead, priorities emerged inductively from participant discussions.*

References to diseases for which participants preferred to have RDTs for quick results and for self-testing were also coded. Relevant excerpts from the transcripts relating to disease priorities and RDT preferences were extracted for synthesis. Using thematic analysis, these codes were grouped into broader themes. Participants did not review the analysis.

This has been clarified in the manuscript on page 8, lines 178-187.

Reviewer 1 comment: 3) The transcript segments included in the results are not particularly helpful. I recommend including only key quotes from participants that illustrate the main themes that emerged from the analysis; full interview segments could be included in a supplement. For example, this approach was executed well on page 11, lines 27-47.

Author response: *We have revised the results to include only key illustrative quotes. Longer excerpts have been moved to a supplementary file 2.*

Reviewer 1 comment: Introduction: It would be helpful to add details regarding which RDTs for high-burden diseases are currently available but require resources for rollout, and which are still in development.

Author response: *The introduction has been revised to include details regarding RDTs that are available and still in development.*

Reviewer 1 comment: Page 7, lines 10-11: Please add appropriate references for the DASH study.

Author response: *This has been revised in the manuscript, page 6, lines 118-126. The DASH protocol was not published. The main findings from the broader DASH study are yet to be published.*

Reviewer 1 comment: Methods section: It would be helpful to include in parentheses the initials of the co-authors who contributed to the different components of data collection and analysis.

Author response: *We have added initials in parenthesis to indicate contributions of co-authors to data collection and analysis.*

Table 1: Percentages are missing for the study population breakdown by age group. The table percentages have been updated on page 9.

Reviewer 1 comment: Results section: It would be helpful to know how the demographics of the participants compare to the broader population. For example, is it expected that 66.7% are unemployed (and are these mostly caregivers?)?

Author response: *We appreciate the need for deeper demographic context. However, our study's data collection on employment was limited. We did not collect additional data on the reasons for unemployment or on participants' caregiver status. While unemployment is high in South Africa, especially in rural provinces such as KwaZulu-Natal, the unemployment rate amongst the study sample was notably higher than the national and provincial average in the same year. This is discussed in 13, lines 335 – 338.*

RESPONSES TO REVIEWED 2 COMMENTS

Reviewer 2 comment: 1. Writing Style and Language: The manuscript would benefit from a thorough review of grammar, punctuation, and sentence structure to enhance readability and flow.

Author response: *The manuscript has been revised for grammar, punctuation and clarity. The authors reviewed the manuscript.*

Reviewer 2 comment: 2. References: Please carefully review and verify all references. Additionally, the reference list includes works as far back as 2002. While some foundational references are acceptable, please consider updating citations with more recent literature to reflect the current state of research and policy, especially in this context.

Author response: *The reference list has been reviewed and updated with more recent literature as far as possible.*

VERSION 2 - REVIEW

**Reviewer**	**1**
**Name**	**Wiens, Kirsten E.**
**Affiliation**	**Temple University**
**Date**	**02-Oct-2025**
**COI**	

I thank the authors for their thorough responses to my review, and I have no further comments.
